# Serum uric acid level and prognosis of acute coronary syndrome: a systematic review and dose-response meta-analysis

**DOI:** 10.3389/fcvm.2025.1670418

**Published:** 2026-01-09

**Authors:** Zongle Sun, Ju Hui, Yan Wang, Jiankang Wang, Rong He, Lina Lyu, Yingzi Cui, Jiajuan Guo

**Affiliations:** 1 Changchun University of Chinese Medicine, Changchun, Jilin, China; 2 The Fifth People’s Hospital of Qingdao, Qingdao, Shandong, China; 3 Affiliated Hospital to Changchun University of Chinese Medicine, Changchun, Jilin, China; 4 Weifang Hospital of Traditional Chinese Medicine, Weifang, Shandong, China

**Keywords:** serum uric acid, acute coronary syndrome, prognosis, systematic review, meta-analysis

## Abstract

**Background:**

This study aims to perform a systematic review and meta-analysis evaluating the association between serum uric acid (SUA) concentrations and clinical outcomes in individuals diagnosed with acute coronary syndrome (ACS).

**Methods:**

PubMed, Web of Science, Embase, and the Cochrane Library were searched up to March 2025. Stata (15.1) was employed to assess heterogeneity, perform sensitivity analyses, evaluate publication bias, and execute subgroup analyses.

**Results:**

A total of 40 cohort studies involving 105,609 ACS patients were included. The results showed that patients with high serum uric acid (HSUA) had significantly higher risks of all-cause mortality [Hazard Ratio [HR] = 1.81, 95% confidence interval [CI]: 1.47–2.22, *p* < 0.001; Odds Ratio [OR] = 1.97, 95% CI: 1.29–2.99, *p* < 0.05], major adverse cardiovascular events (MACE) (HR = 1.40, 95% CI: 1.15–1.71, *p* < 0.05; OR=2.25, 95% CI: 1.73–2.92, *p* < 0.001), cardiovascular mortality (HR = 2.58, 95% CI: 1.67–3.98, *p* < 0.001), stroke [risk ratio (RR) = 1.27, 95% CI: 1.08–1.48, *p* < 0.05], and heart failure (RR = 1.90, 95% CI: 1.72–2.11, *p* < 0.001) compared to those with non-HSUA level. However, there was no significant effect on the risk of revascularization (RR = 1.09, 95% CI: 0.80–1.47, *p* = 0.594). Subgroup analyses suggested that follow-up time, type of ACS, treatment methods, and region might influence the observed associations. Additionally, SUA level was also nonlinearly related to all-cause and cardiovascular mortality.

**Conclusion:**

HSUA level is strongly associated with poor clinical outcomes in ACS patients, including mortality and major cardiovascular events. Given the nonlinear relationship with mortality, SUA could serve as a potentially valuable clinical marker. However, further multicenter studies are needed to confirm these findings.

## 1 Introduction

Every year, millions of people die from acute coronary syndrome (ACS). This makes the search for reliable prognostic markers more important than ever. Unstable angina, non-ST-segment elevation myocardial infarction (NSTEMI), and ST-segment elevation myocardial infarction (STEMI) are all types of ACS. It is still a major cause of illness and death around the world (1). The risks of death, heart failure, and recurrent events are high for survivors. There has been advancement in reperfusion treatments and instruments such as the GRACE score (2). However, because of unresolved residual risks, many patients continue to fall through the cracks. This emphasizes the necessity of easily accessible and adjustable biomarkers (3). Serum uric acid (SUA), traditionally regarded as a culprit in gout due to its role as a byproduct of purine metabolism, has now emerged as a multifaceted player in cardiovascular pathology. It exerts effects through mechanisms such as NLRP3 inflammasome activation, promotion of oxidative stress, and endothelial dysfunction (4, 5). High serum uric acid (HSUA) levels are linked to worse outcomes in ACS, such as bigger infarcts, worse left ventricular function, and higher death rates (6). Some studies, on the other hand, don't find a link (7). This makes people wonder if SUA is a cause of ACS.

There were problems with previous meta-analyses (8), such as small sample sizes, big differences in the makeup of the populations studied, and not enough research into dose-response or subgroup effects. The number of people around the world with hyperuricemia is going up (9). We need to take a closer look at the role of SUA in predicting the outcome of ACS. In this systematic review and meta-analysis of cohort studies with more than 100,000 ACS patients, we first looked at the combined risks of death from any cause, death from heart disease, and major adverse cardiovascular events (MACE). Next, we looked at how different doses affected different groups of people based on the type of ACS, how long the follow-up lasted, and where the people lived. The goal of this study is to give stronger clinical evidence about how well SUA predicts the outcome in ACS patients and to support more personalized care.

## 2 Methods

### 2.1 Literature search

This PRISMA-compliant meta-analysis was prospectively registered on the PROSPERO international prospective register of systematic reviews (Registration ID: CRD420251013025) (10). The Cochrane Library, Embase, Web of Science, and PubMed were retrieved between the database's creation and March 2025. Among the search terms were unstable angina, myocardial infarction, acute coronary syndrome, and uric acid. Supplementary Table S1 provides more search details.

### 2.2 Screening criteria

Two researchers, Zongle Sun and Ju Hui, first checked titles and abstracts and then analyzed the full text of the literature. Any doubts were resolved by discussion with Yan Wang. The screening followed the following rules.

### 2.3 Inclusion criteria

The following requirements were satisfied by eligible studies: (i) patients diagnosed with ACS; (ii) the exposed group included ACS patients with high serum uric acid (HSUA); (iii) the control group included ACS patients with non-HSUA; (iv) primary outcomes included all-cause mortality, cardiovascular mortality, MACE, stroke, revascularization, and heart failure; and (v) cohort design.

### 2.4 Exclusion criteria

The following studies were excluded: (i) animal experiments; (ii) meta, review, meeting, abstract, case report, guideline; (iii) duplicate articles; (iv) no available data for analysis.

### 2.5 Data extraction

Zongle Sun and Ju Hui, the authors, will filter articles based on the chosen inclusion criteria. Discussions with Yan Wang, the third researcher, about the final inclusion criteria will settle any disagreements. Author names, publication year, country of origin, study design, sample size, demographic characteristics (age, gender), follow-up time, data analysis methods, classification of SUA, type of ACS, treatment methods, and outcomes are among the information that will be included.

### 2.6 Definition of outcome indicators

The definition of HSUA varied among the included studies. In most studies, HSUA was defined as SUA levels >7.0 mg/dL in men and >6.0 mg/dL in women, whereas others used study-specific cut-off values or quantile-based categories. To maintain consistency with the original data, we adopted the definitions reported by each study and summarized them in Supplementary Table S2.

The definition of MACE varied among studies. Most defined MACE as a composite of cardiovascular death, non-fatal myocardial infarction, and non-fatal stroke, while some also included revascularization, heart failure, or unstable angina. To maintain consistency and transparency, the original definitions were retained and summarized in Supplementary Table S2.

Short term outcome indicators: (i) all-cause mortality within 30 days and cardiovascular mortality within 30 days, both of which include deaths occurring during hospitalization; (ii) MACE within 30 days; (iii) other adverse clinical outcomes within 30 days, such as revascularization, stroke, and heart failure.Middle to long term outcome indicators: (i) all-cause mortality at 6 months or longer and cardiovascular mortality at 6 months or longer; (ii) MACE at 6 months or longer; (iii) other adverse clinical outcomes at 6 months or longer, such as revascularization, stroke, and heart failure. More details are provided in Supplementary Table S2.

### 2.7 Quality evaluation

Two reviewers independently assessed study quality using a domain-adapted Newcastle–Ottawa Scale (NOS) (11); Consultation with a third reviewer was used to settle any disputes. The adaptation follows domain-specific practices reported in the literature and prespecifies thresholds for exposure ascertainment, outcome assessment, adequacy of follow-up, and comparability. Full scoring criteria are provided in the Supplementary Tables S3 and S4.

### 2.8 Statistical analysis

Stata (15.1) software was used to analyze the data. A random-effects model with the DerSimonian and Laird weighted technique was used to conduct the meta-analysis (12). To address variations in reported effect measures across studies, analyses were stratified by the type of effect measure: hazard ratio (HR) for time-to-event outcomes and odds ratio (OR) or relative risk (RR) for binary outcomes. Where studies reported RR or OR, these were pooled separately after transforming RR to OR using standard methods to ensure consistency within that stratum; HR were not transformed or pooled with OR/RR due to their distinct statistical interpretations and underlying assumptions. Separate forest plots were generated for each stratum (e.g., one for HR and one for OR) to estimate overall effects without combining incompatible measures (13). Meta-analysis of the studies was depicted as forest plots to estimate the overall RR, OR and HR. *I*^2^ statistics were used to assess study heterogeneity and were classed as follows: Because of the significant heterogeneity shown by *I*^2^ ≥ 50%, a random-effects model was used to account for between-study variation (14). Sensitivity analysis (leave-one-out method) identified potential sources of heterogeneity. Subgroup analyses stratified studies by control group type. For analyses with *I*^2^ < 50%, a fixed-effects model was applied, assuming a common effect size across studies. Methods for evaluating potential bias in publications included Egger's test and the funnel plot (15). When conducting two-tailed tests, statistical significance was defined as a two-tailed *p*-value < 0.05 in all analyses. We conducted a dose-response analysis using a recognized technique in order to thoroughly describe the dose-response relationship (16–18). For this method, at least three quantitative exposure categories have to have their total number of participants and cases, HR estimates, and variance estimates. The associated risk estimate for each study was based on the median/mean uric acid levels for each category. For categories lacking reported median/mean UA values, the central tendency was estimated by taking the midpoint of the upper and lower category boundaries. The interval's width was assumed to be identical to that of the nearest category if either the highest or lowest category was open-ended (19). When the reference category utilized in the analysis was not the lowest group, risk estimates were converted using the Hamling et al. (20) technique. In order to investigate the possible correlation between the risk of cardiovascular events and uric acid levels, we used restricted cubic splines to model exposure levels. Three knots were positioned at the 25th, 50th, and 75th percentiles of the exposure distribution. The null hypothesis that the coefficient of the second spline term was equal to zero was tested in order to determine nonlinearity. Every statistical test had a significance threshold of *p* < 0.05 and was two-sided.

## 3 Results

### 3.1 Literature search and screening process

**Figure 1:**
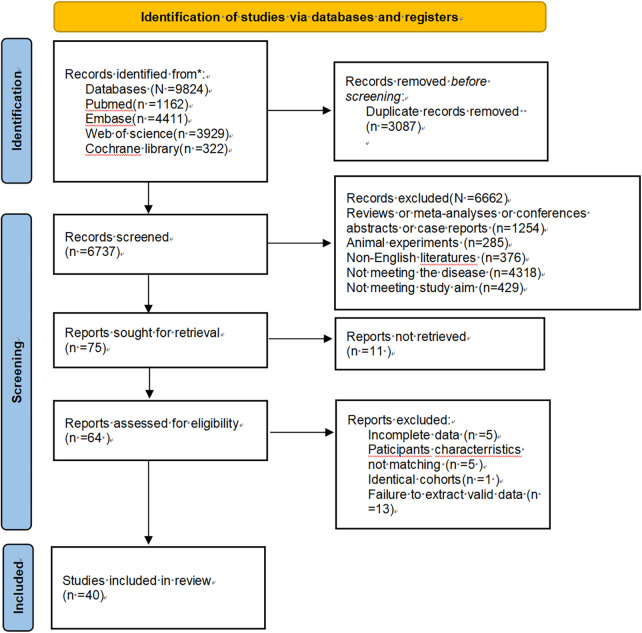
Flowchart of the preferred reporting items for systematic reviews and meta-analyses.

The literature search yielded 9,824 potentially relevant articles. Automated deduplication reduced this to 3,087 unique publications. Screening of titles and abstracts further narrowed the set to 64 articles, which were then subjected to full-text review. Ultimately, 40 articles were included in the analysis. The details can be found in Figure 1.

### 3.2 Methodological quality and baseline characteristics of selected studies

A total of 40 (6, 7, 21–58) studies, encompassing various countries and regions, were included. These comprised both prospective and retrospective cohort studies conducted between 2005 and 2025, involving 105,609 ACS patients with sample sizes ranging from 184 to 27,959. Participant demographics varied in age, gender, ACS type, follow-up time, data analysis methods, classification of SUA, and treatment methods. All-cause mortality, cardiovascular mortality, MACE, heart failure, stroke, and revascularization were the main outcomes evaluated, as indicated in Table 1 and Supplementary Table S2. Subgroup analysis will be conducted from four aspects: follow-up time, type of ACS, treatment methods, and region. Quality assessment using the NOS demonstrated the following score distribution among included studies: 36 studies ≥6 points, 3 studies with 5 points, and 1 study at 4 points (Supplementary Table S4).

**Table 1:** Baseline characteristics.

Author	Publication year	Country	Study design	Sample size	Age	Gender (male/female)	Data analysis methods	Type of ACS	Follow-up time	Treatment methods	Outcome
Akpek et al. (21)	2011	Turkey	Prospective cohort study	289	60.00	221/67	Multivariate analysis	STEMI	1 year	PCI	Mace/Revascularization
Basar et al. (22)	2011	Turkey	Retrospective cohort study	185	58.74	148/37	Multivariate analysis	STEMI	In-hospital period	PCI	All-Cause Mortality
Chen et al. (23)	2012	China	Retrospective cohort study	502	61.27	417/85	Multivariate analysis	STEMI	In-hospital period/24.3 months/5 years	PCI/non-PCI	Stroke/Heart failure
Kaya et al. (24)	2012	Turkey	Prospective cohort study	2,249	57.14	1,853/396	Multivariate analysis	STEMI	average of 2.8 years	PCI	Cardiovascular Mortality/Mace/Revascularization/Stroke/Heart failure
Krishnan et al. (25)	2012	America	Prospective cohort study	4,352	54.90	3,877/475	Univariate Analysis	STEMI/NSTEMI	1 year	PCI/non-PCI	All-Cause Mortality/Cardiovascular Mortality
Ndrepepa et al. (26)	2012	Germany	Prospective cohort study	5,124	67.87	3,762/1,362	Multivariate analysis	ACS	In-hospital period	PCI	All-Cause Mortality
Omidvar et al. (27)	2012	Iran	Prospective cohort study	184	58.00	129/55	Multivariate analysis	STEMI	In-hospital period	PCI/non-PCI	All-Cause Mortality
Wang et al. (28)	2012	China	Prospective cohort study	276	56.41 ± 11.22	221/55	Multivariate analysis	STEMI	3.5 years	PCI	No-reflow Phenomenon
Levantesi et al. (29)	2013	Italy	Retrospective cohort study	10,840	59.30	9,247/1,593	Multivariate analysis	STEMI/NSTEMI	1 year	PCI/non-PCI	All-Cause Mortality/Cardiovascular Mortality
Timóteo et al. (30)	2013	Portugal	Retrospective cohort study	683	64.00	471/212	Multivariate analysis	ACS	6 months	PCI/non-PCI	All-Cause Mortality
Akgul et al. (31)	2014	Turkey	Prospective cohort study	434	55.40	341/93	Multivariate analysis	STEMI	In-hospital period	PCI/non-PCI	All-Cause Mortality/Revascularization/Stroke/Heart failure
Gazi et al. (32)	2014	Turkey	Retrospective cohort study	586	61.02	467/119	Multivariate analysis	STEMI	In-hospital period	PCI/non-PCI	Cardiovascular Mortality/Heart failure
Karim et al. (33)	2015	Indonesia	Retrospective cohort study	251	57.10	169/82	Univariate Analysis	ACS	1 year	PCI/non-PCI	Mace/Revascularization
Lazzeri et al. (34)	2015	Italy	Prospective cohort study	329	77.20	177/152	Multivariate analysis	STEMI	3 years	PCI	All-Cause Mortality
Von Lueder et al. (35)	2015	Multiple countries	Retrospective cohort study	12,677	65.25	9,065/3,612	Multivariate analysis	STEMI/NSTEMI	20 months	PCI/non-PCI	All-Cause Mortality/Cardiovascular Mortality
Hajizadeh et al. (36)	2016	Iran	Prospective cohort study	608	62.60	437/171	Multivariate analysis	STEMI	In-hospital period	PCI/non-PCI	Stroke
Ranjith et al. (40)	2016	South Africa	Retrospective cohort study	2,683	57.10	1,740/943	Multivariate analysis	STEMI/NSTEMI	30 days/1 year	PCI/non-PCI	Cardiovascular Mortality/Mace/Stroke/Heart failure
Liu et al. (7)	2017	China	Retrospective cohort study	944	57.69	831/113	Multivariate analysis	STEMI	In-hospital period	PCI	All-Cause Mortality
Magnoni et al. (37)	2017	Italy	Retrospective cohort study	1,548	62.71	1,121/427	Multivariate analysis	ACS	In-hospital period	PCI/non-PCI	All-Cause Mortality
Morn et al. (38)	2017	Mexico	Retrospective cohort study	795	59.00	668/127	Multivariate analysis	STEMI	The median follow-up period of 365 days	PCI/non-PCI	All-Cause Mortality/Stroke/Heart failure
Pagidipati et al. (39)	2017	Multiple countries	Retrospective cohort study	27,959	63.35	20,068/7,891	Univariate Analysis/Multivariate analysis	ACS	2 years	PCI/non-PCI	Stroke
Kobayashi et al. (41)	2018	Japan	Retrospective cohort study	1,308	66.62	1,042/266	Univariate Analysis	ACS	2 years	PCI/non-PCI	Cardiovascular Mortality
Kobayashi et al. (42)	2018	Japan	Retrospective cohort study	1,114	66.44	995/119	Multivariate analysis/propensity score adjusted	ACS	The median follow-up period of 5.5 ± 2.9 years	PCI/non-PCI	Cardiovascular Mortality
Tscharre et al. (44)	2018	Austria	Prospective cohort study	1,215	62.90	805/408	Multivariate analysis	ACS	5 years	PCI/non-PCI	Cardiovascular Mortality/Mace
Lopez et al. (43)	2018	Spain	Prospective cohort study	1,119	68.10	830/289	Multivariate analysis	STEMI/NSTEMI	The median follow-up period of 246.31 ± 49.16 days	PCI/non-PCI	All-Cause Mortality/Cardiovascular Mortality/Mace
Ye et al. (45)	2018	China	Retrospective cohort study	2,296	60.09	1,558/738	Multivariate analysis	ACS	In-hospital period/1 year	PCI	All-Cause Mortality
Tai et al. (48)	2019	China	Retrospective cohort study	711	78.12	438/273	Multivariate analysis	STEMI/NSTEMI	The median follow-up of 2.3 ± 1.0 years	PCI/non-PCI	All-Cause Mortality/Revascularization/Stroke/Heart failure
Guo et al. (46)	2019	China	Prospective cohort study	1,005	61.82	849/156	Univariate Analysis	STEMI/NSTEMI	1 year	PCI	All-Cause Mortality
Mandurino et al. (51)	2020	Italy	Prospective cohort study	2,369	63.35	1,840/529	Multivariate analysis	STEMI	In-hospital period	PCI	All-Cause Mortality
Centola et al. (47)	2020	Italy	Retrospective cohort study	1,088	68.7 ± 13.36	830/258	Multivariate analysis	ACS	The median follow-up of 41.7 months	PCI/non-PCI	All-Cause Mortality
Ma et al. (50)	2021	China	Prospective cohort study	1,179	55.70	867/312	Multivariate analysis	STEMI/NSTEMI	30 months	PCI/non-PCI	Mace/Revascularization/Stroke/Heart failure
Mohammed et al. (52)	2021	China	Prospective cohort study	249	62.67	127/122	propensity score adjusted	STEMI/NSTEMI	The median follow-up of 5.02 (3.07, 7.55) years	PCI/non-PCI	Mace/Stroke/Heart failure
Kim et al. (53)	2022	Korea	Retrospective cohort study	5,888	64.00	4,141/1,744	Multivariate analysis	STEMI/NSTEMI	In-hospital period	PCI	All-Cause Mortality/Cardiovascular Mortality/Revascularization/Stroke/Heart failure
Nakahashi et al. (55)	2022	Japan	Retrospective cohort study	989	68.00	763/226	Multivariate analysis	STEMI/NSTEMI	5 years	PCI	All-Cause Mortality
Tang et al. (56)	2022	China	Prospective cohort study	1,448	-	1,238/210	Multivariate analysis	STEMI	The median of 4 years	PCI	All-Cause Mortality/Cardiovascular mortality/Mace/Revascularization/Stroke
Nakahashi et al. (54)	2022	America	Retrospective cohort study	1,068	69.00	794/74	Univariate Analysis	ACS	3 years	PCI	All-Cause Mortality
Dyrbuś et al. (49)	2023	Poland	Retrospective cohort study	2,824	66.12	1,844/980	Multivariate analysis	ACS	1 year	PCI/non-PCI	All-Cause Mortality
Liang et al. (57)	2023	China	Retrospective cohort study	1,396	63.90	1,104/292	Multivariate analysis	STEMI	1 year	PCI/non-PCI	All-Cause Mortality
Nie et al. (6)	2024	China	Retrospective cohort study	4,319	63.07	3,144/1,175	Multivariate analysis	STEMI/NSTEMI	The median follow-up of 64 (46, 79) months	PCI/non-PCI	All-Cause Mortality/Cardiovascular Mortality
Li et al. (58)	2025	China	Retrospective cohort study	526	81.35	327/199	Data analysis methods	ACS	Follow-up time	PCI/non-PCI	Mace

### 3.3 All-cause mortality

**Figure 2:**
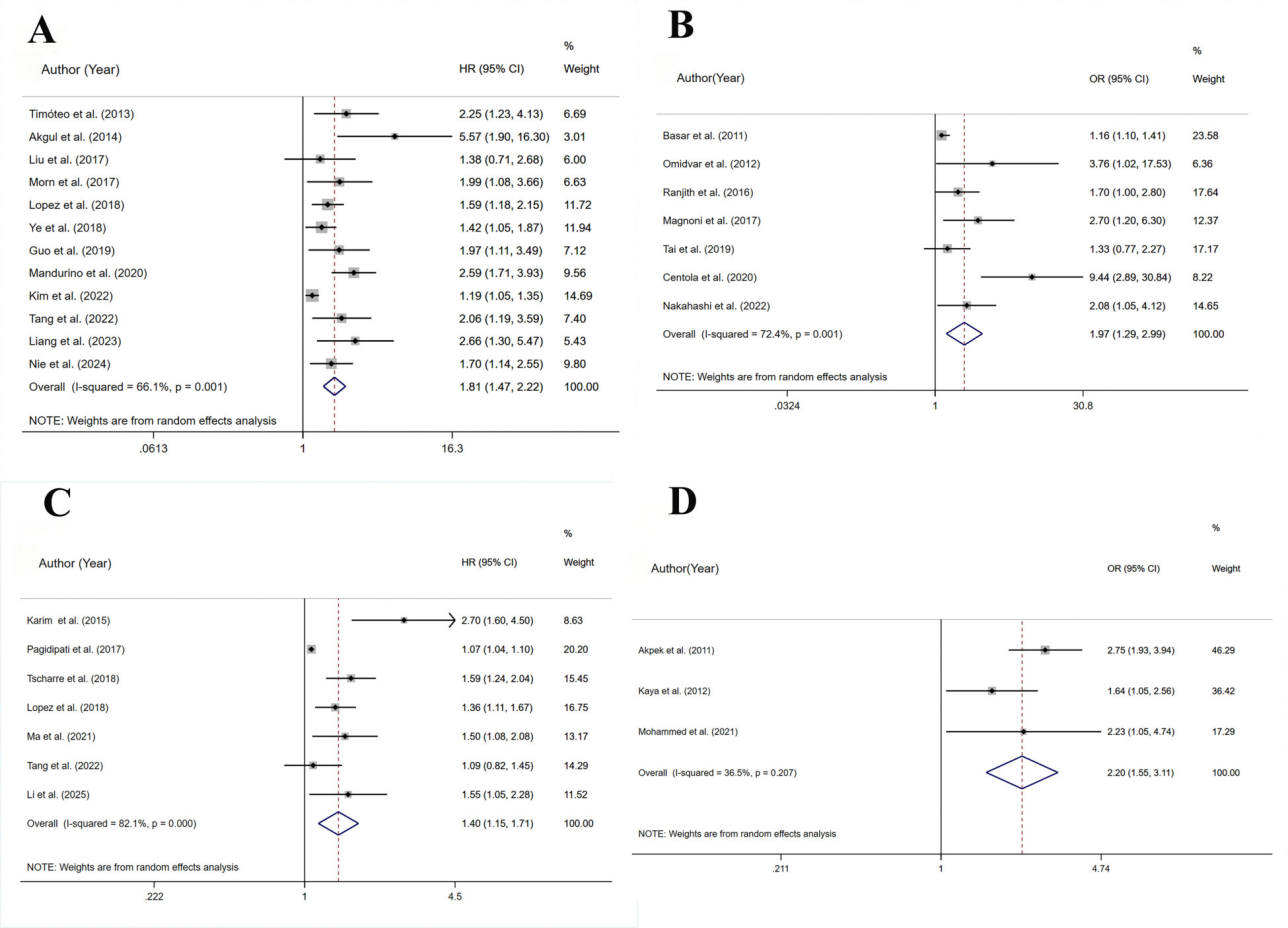
Meta-analysis on the prognosis of ACS patients with HSUA and Non-HSUA groups. **(A)** All-cause mortality(HR). **(B)** All-cause mortality(OR). **(C)** MACE(HR). **(D)** MACE(OR).

A total of 12 studies assessed all-cause mortality(HR), involving 22,696 patients. Heterogeneity analysis (*I*^2^ = 66.1%, *p* < 0.05) showed significant heterogeneity. The risk of all-cause mortality was significantly higher for the HSUA group than for the non-HSUA group (HR = 1.81, 95% CI: 1.47–2.22, *p* < 0.001) (Figure 2A and Supplementary Table S5). Subgroup analyses revealed that across multiple clinical categories, the HSUA group had a higher all-cause mortality rate than the non-HSUA group: Middle to long follow-up (11 studies; HR = 1.80, 95% CI: 1.44–2.23, *p* < 0.001), Short follow-up (2 studies; HR = 1.69, 95% CI: 1.08–2.64, *p* < 0.001), STEMI (6 studies; HR = 2.07, 95% CI: 1.59–2.70, *p* < 0.001), ACS (3 studies; HR = 1.84, 95% CI: 1.23–2.76, *p* < 0.05), STEMI/NSTEMI (3 studies; HR = 1.43, 95% CI: 1.08–1.89, *p* < 0.05), Percutaneous coronary intervention (PCI) (6 studies; HR = 1.64, 95% CI: 1.24–2.17, *p* < 0.05), PCI/non-PCI (6 studies; HR = 1.95, 95% CI: 1.53–2.50, *p* < 0.001), Europe (4 studies; HR = 2.28, 95% CI: 1.53–3.40, *p* < 0.001), America (1 study; HR = 1.99, 95% CI: 1.08–3.66, *p* < 0.05),and Asia (7 studies; HR = 1.55, 95% CI: 1.25–1.92, *p* < 0.001) (Supplementary Figure S1 and Supplementary Table S5). The Middle to long follow-up, Short follow-up, STEMI, ACS, STEMI/NSTEMI, PCI/non-PCI, Europe, Asia subgroups showed less heterogeneity (Supplementary Figure S1 and Supplementary Table S5).

A total of 7 studies assessed all-cause mortality(OR), involving 7,467 patients. Heterogeneity analysis (*I*^2^ = 72.4%, *p* < 0.05) showed significant heterogeneity. The risk of all-cause mortality was significantly higher for the HSUA group than for the non-HSUA group (OR = 1.97, 95% CI: 1.29–2.99, *p* < 0.05) (Figure 2B and Supplementary Table S5). Subgroup analyses revealed that across multiple clinical categories, the HSUA group had a higher all-cause mortality rate than the non-HSUA group: Middle to long follow-up (2 studies; OR = 1.17, 95% CI: 1.03–1.32, *p* < 0.05), Short follow-up (5 studies; OR = 2.67, 95% CI: 1.60–4.45, *p* < 0.001), ACS (2 studies; OR = 4.69, 95% CI: 1.39–15.85, *p* < 0.05), STEMI/NSTEMI (3 studies; OR = 1.63, 95% CI: 1.17–2.26, *p* < 0.05), PCI/non-PCI (5 studies; OR = 2.43, 95% CI: 1.37–4.31, *p* < 0.05),Asia (3 studies; OR = 1.55, 95% CI: 1.10–2.74, *p* < 0.05), and Africa (1 study; OR = 1.70, 95% CI: 1.02–2.84, *p* < 0.05) (Supplementary Figure S1 and Supplementary Table S5). No notable differences in all-cause mortality were detected among the subgroups of Middle to long follow-up, STEMI, PCI, and Europe (Supplementary Figure S1 and Supplementary Table S5). The Middle to long follow-up, Short follow-up, STEMI, ACS, STEMI/NSTEMI, PCI, Asia subgroups showed less heterogeneity (Supplementary Figure S1 and Supplementary Table S5).

### 3.4 MACE

A total of 7 studies assessed MACE(HR), involving 33,697 patients. Heterogeneity analysis (*I*^2^ = 82.1%, *p* < 0.001) showed significant heterogeneity. In comparison to the non-HSUA group, the HSUA group had a significantly elevated risk of MACE (HR = 1.40, 95% CI: 1.15–1.71, *p* < 0.05) (Figure 2C and Supplementary Table S5). The subgroup demonstrated that the HSUA group had significantly higher MACE risk compared with the non-HSUA group across multiple categories: ACS (4 studies; HR = 1.55, 95% CI: 1.08–2.22, *p* < 0.05),STEMI/NSTEMI (2 studies; HR = 1.40, 95% CI: 1.18–1.66, *p* < 0.001), Asia (4 studies; HR = 1.54, 95% CI: 1.11–2.14, *p* < 0.05), Others (3 studies; HR = 1.29, 95% CI: 1.00–1.67, *p* < 0.05). No notable changes were detected in the “STEMI” groupings (Supplementary Figure S1 and Supplementary Table S5). Reduced heterogeneity was observed in the STEMI/NSTEMI category (Supplementary Figure S1 and Supplementary Table S5).

A total of 3 studies assessed MACE(OR), involving 2,787 patients. Heterogeneity analysis (*I*^2^ = 35.5%, *p* = 0.207) showed low heterogeneity. In comparison to the non-HSUA group, the HSUA group had a significantly elevated risk of MACE (OR = 2.25, 95% CI: 1.73–2.92, *p* < 0.001) (Figure 2D and Supplementary Table S5). The subgroup analysis demonstrated that the HSUA group had significantly higher MACE risk compared with the non-HSUA group across different follow-up time: Middle to long follow-up (2 studies; OR = 1.78, 95% CI: 1.21–2.61, *p* < 0.05), Short follow-up (2 studies; OR = 2.51, 95% CI: 1.86–3.89, *p* < 0.001). Reduced heterogeneity was observed in both subgroups (Supplementary Figure S1 and Supplementary Table S5).

### 3.5 Cardiovascular mortality

**Figure 3:**
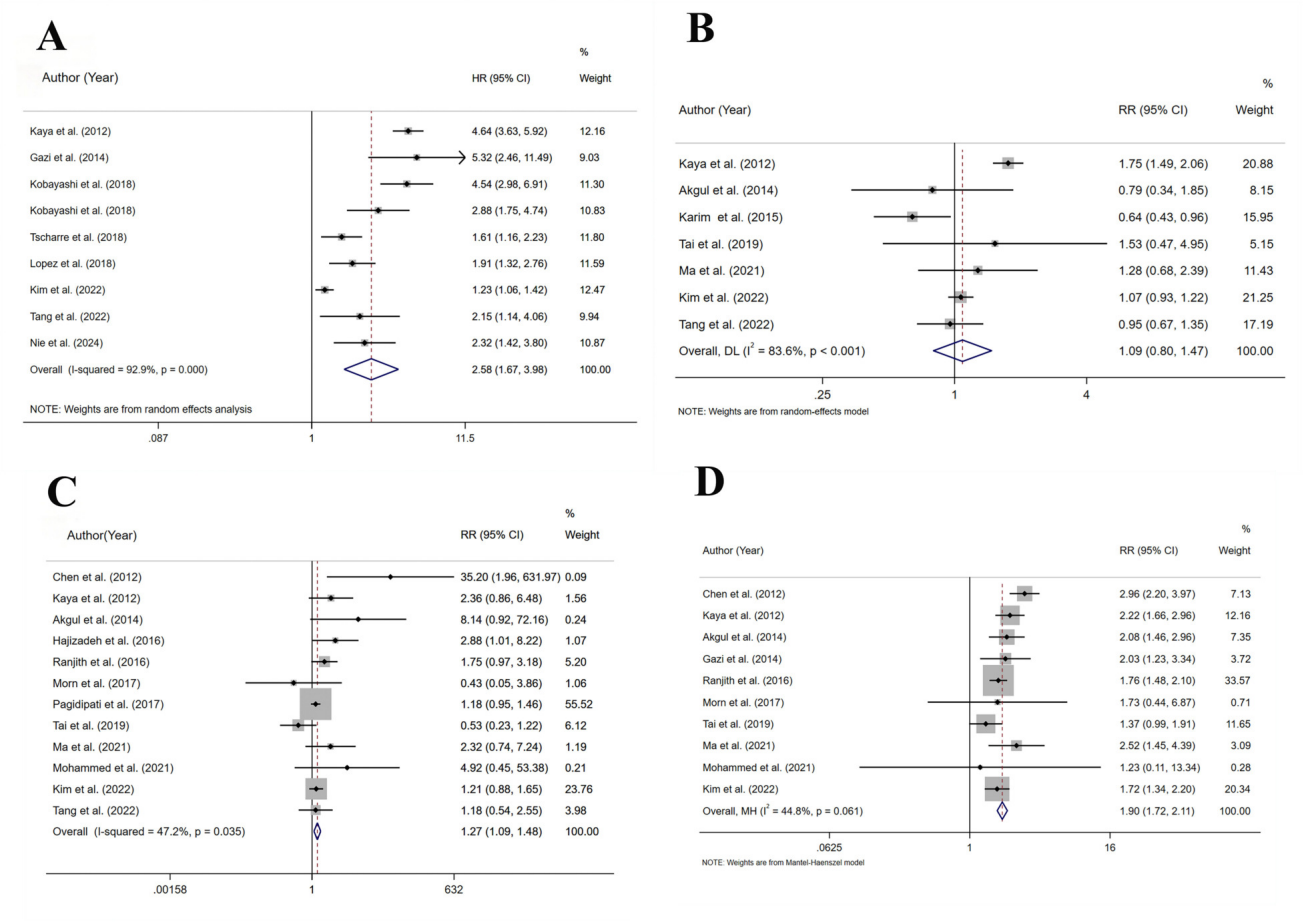
Meta-analysis on the prognosis of ACS patients with HSUA and Non-HSUA groups. **(A)** Cardiovascular mortality. **(B)** Revascularization. **(C)** Stroke. **(D)** Heart failure.

A total of 9 studies assessed cardiovascular mortality, involving 19,246 patients. Heterogeneity analysis (*I*^2^ = 92.9%, *p* < 0.001) showed significant heterogeneity. The risk of cardiovascular mortality (HR = 2.58, 95% CI: 1.67–3.98, *p* < 0.001) substantially increased in the HSUA group compared to the non-HSUA group (Figure 3A and Supplementary Table S5).

The subgroup analysis revealed that the HSUA group exhibited a higher risk of cardiovascular mortality compared to the non-HSUA group in all evaluated categories: STEMI (3 studies; HR = 3.84, 95% CI: 2.34–6.32, *p* < 0.001), ACS (3 studies; HR = 2.73, 95% CI: 1.43–5.23, *p* < 0.05), STEMI/NSTEMI (3 studies; HR = 1.68, 95% CI: 1.11–2.53, *p* < 0.001), PCI/non-PCI (6 studies; HR = 2.68, 95% CI: 1.84–3.89, *p* < 0.001), Europe (3 studies; HR = 3.50, 95% CI: 1.78–6.88, *p* < 0.001), and Asia (5 studies; HR = 2.37, 95% CI: 1.33–4.24, *p* < 0.05). No substantial differences were observed in the PCI subgroup (Supplementary Figure S1 and Supplementary Table S5). The STEMI subgroups exhibited reduced heterogeneity (Supplementary Figure S1 and Supplementary Table S5).

### 3.6 Revascularization

A total of 7 studies assessed revascularization, involving 19,960 patients. Heterogeneity analysis (*I*^2^ = 83.6%, *p* < 0.001) showed significant heterogeneity. In comparison to the non-HSUA group, the HSUA group presented no statistically notable elevation in the risk of revascularization (RR = 1.09, 95% CI: 0.80–1.47, *p* = 0.594) (Figure 3B and Supplementary Table S5).

In the short-term (RR = 0.67, 95% CI: 0.46–0.96, *p* < 0.05) and ACS (RR = 0.64, 95% CI: 0.43–0.96, *p* < 0.001) subgroups, the HSUA group showed a significantly lower revascularization risk compared to the non-HSUA group. No notable differences in revascularization risk were detected among the subgroups of Middle to long follow-up, STEMI, STEMI/NSTEMI, Europe, and Asia (Supplementary Figure S1 and Supplementary Table S5). Lower heterogeneity was found in the Short follow-up, ACS, STEMI/NSTEMI, and Asia subgroups (Supplementary Figure S1 and Supplementary Table S5).

### 3.7 Stroke

A total of 12 studies assessed stroke, involving 44,705 patients. Heterogeneity analysis (I^2^ = 47.2%, *p* = 0.035) showed moderate heterogeneity. In comparison to the non-HSUA group, the HSUA group presented a significantly elevated risk of stroke (RR = 1.27, 95% CI: 1.08–1.48, *p* < 0.05) (Figure 3C and Supplementary Table S5).

The subgroup analysis results indicated that the HSUA group had a significantly higher risk of stroke compared with the non-HSUA group in numerous categories: Short follow-up (7 studies; RR = 1.92, 95% CI: 1.10–3.35, *p* < 0.05), STEMI (6 studies; RR = 2.30, 95% CI: 1.05–5.01, *p* < 0.05), PCI/non-PCI (9 studies; RR = 1.27, 95% CI: 1.06–1.53, *p* < 0.05), and Europe (2 studies; RR = 2.96, 95% CI: 1.16–7.53, *p* < 0.05). No substantial differences in stroke risk were noted among the subgroups of Middle to long, ACS, STEMI/non-STEMI, PCI, Asia, and “Others” (Supplementary Figure S1 and Supplementary Table S5). Lower heterogeneity was found in the Middle to long follow-up, STEMI, ACS, STEMI/NSTEMI, PCI/non-PCI, Europe, and “Others” subgroups (Supplementary Figure S1 and Supplementary Table S5).

### 3.8 Heart failure

A total of 10 studies assessed heart failure, involving 15,276 patients. Heterogeneity analysis (*I*^2^ = 44.8%, *p* = 0.061) showed moderate heterogeneity. In comparison to the non-HSUA group, the HSUA group presented a substantially elevated risk of heart failure (RR = 1.90, 95% CI: 1.72–2.11, *p* < 0.001) (Figure 3D and Supplementary Table S5).

Subgroup analysis found that the prospective risk of heart failure in the listed groups of the HSUA group surpassed that of the non-HSUA group: Middle to long follow-up (4 studies; RR = 1.95, 95% CI: 1.64–2.23, *p* < 0.001), Short follow-up (6 studies; RR = 1.88, 95% CI: 1.66–2.12, *p* < 0.001), STEMI (5 studies; RR = 2.32, 95% CI: 1.86–2.74, *p* < 0.001), STEMI/NSTEMI (5 studies; RR = 1.71, 95% CI: 1.51–1.95, *p* < 0.001), PCI (2 studies; RR = 1.90, 95% CI: 1.58–2.29, *p* < 0.001), PCI/non-PCI (8 studies; RR = 1.90, 95% CI: 1.69–2.14, *p* < 0.001), Europe (3 studies; RR = 2.14, 95% CI: 1.61–2.21, *p* < 0.001), Asia (5 studies; RR = 1.89, 95% CI: 1.51–1.95, *p* < 0.001), and “Others” (2 studies; RR = 1.76, 95% CI: 1.48–2.09, *p* < 0.001) (Supplementary Figure S1 and Supplementary Table S5). Lower heterogeneity was found in the Middle to long follow-up, STEMI, STEMI/NSTEMI, PCI, Europe, and “Others” subgroups (Supplementary Figure S1 and Supplementary Table S5).

### 3.9 Dose-response analysis

**Figure 4:**
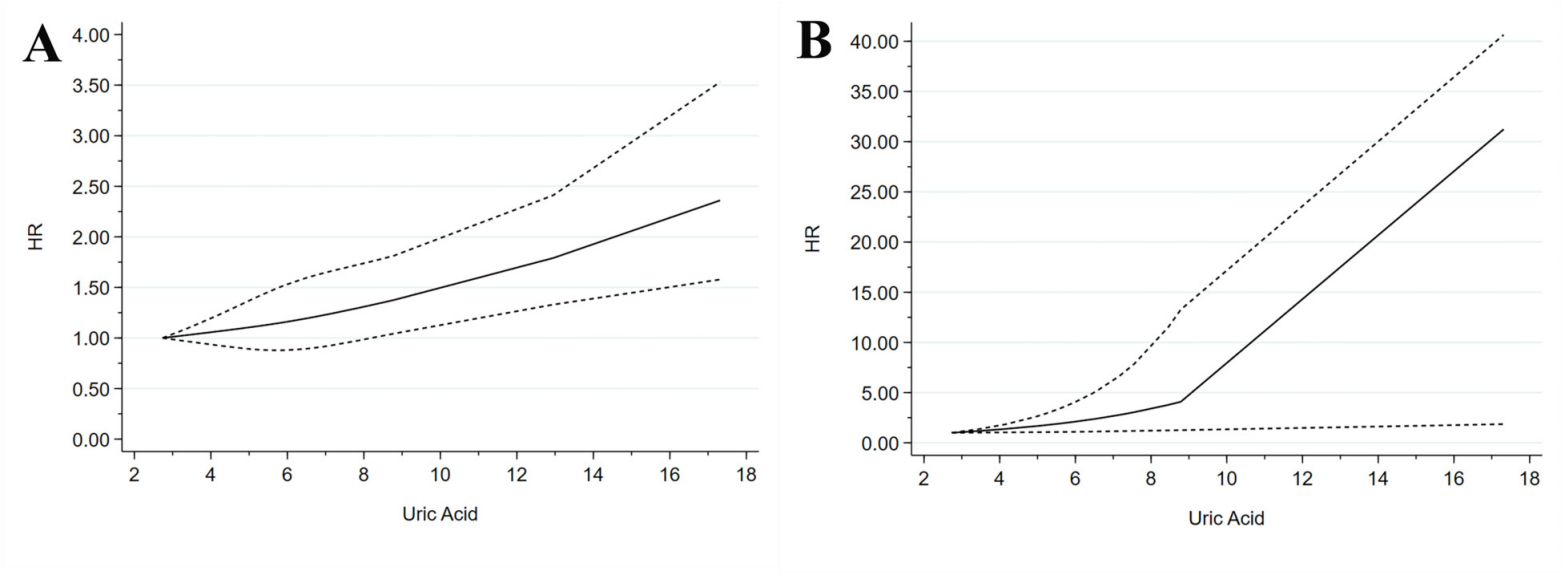
Dose–response meta-analysis between SUA levels and mortality in patients with ACS. **(A)** All-cause mortality. **(B)** Cardiovascular mortality.

The dose-response relationships of SUA concentrations with composite all-cause and cardiovascular mortality outcomes were analyzed. The nonlinear test results indicated that all-cause mortality exhibited a nonlinear trend (*p* = 0.753) (Figure 4A). At 3.978, 5.99, and 8.98 mg/dL, the HR (95% CI) of all-cause mortality were 1.06 (0.95–1.20), 1.16 (0.87–1.52), and 1.41 (1.05–1.84), respectively. Cardiovascular mortality showed a nonlinear trend (*p* = 0.806) (Figure 4B). At 4.00, 8.99, and 12.02 mg/dL, the HR (95% CI) of cardiovascular mortality were 1.28 (0.94–1.69), 4.83 (1.45–14.25), and 12.03 (1.60–23.75), respectively.

### 3.10 Sensitivity analysis and publication bias analysis

By performing a leave-one-out sensitivity analysis, all outcomes demonstrated good robustness. For outcomes with ≥10 studies, funnel plots and Egger's test were conducted to assess publication bias. Egger's test indicated a significant probability of publication bias for the HR for all-cause mortality (*P* < 0.05). The application of the trim-and-fill method to address this bias resulted in a reversal of the estimated impact sizes for the outcome indicators, suggesting that the original analysis results may have been overestimated due to the small study effect or selective publication of positive findings. Consequently, a cautious interpretation is warranted for the outcome. Conversely, the funnel plots and Egger's test results for stroke, and heart failure exhibited no discernible publication bias, indicating that the associations of these outcomes were relatively reliable. The detailed results are illustrated in Supplementary Figure S2.

## 4 Discussion

ACS is still one of the main causes of heart disease and death around the world, so we need reliable prognostic biomarkers to help us better manage patients and improve their outcomes. This systematic review and meta-analysis, which included 40 cohort studies and a total of 105,609 patients, shows that there may be a link between HSUA level and bad clinical outcomes in ACS. This shows that SUA could be a very important tool for figuring out who is at risk.

There is a close two-way link between HSUA and ACS. HSUA can happen because of different problems like metabolism issues, oxidative stress, kidney problems, and long-term inflammation (1, 4, 5). Together, these conditions damage the endothelium, change the structure of blood vessels, and promote clot formation. As a result, they can worsen or sustain the development of ACS. Strong inflammation during ACS can make the body respond more to uric acid crystals. The reaction happens through the NLRP3 inflammasome pathway. This response makes more harmful oxygen substances, stops the blood vessels from making nitric oxide, and causes the cells in the blood vessels to die. These problems make it more likely that plaques in the arteries will break and cause heart attacks to become worse. Furthermore, Rebora et al. suggest that elevated UA may act as an index of peripheral hypoperfusion in ACS patients with acute HF, where it could reflect worsened responses to hypoperfusion (potentially influenced by diuretics), serving as an indirect marker of increased mortality rather than a direct causal factor (59). HSUA can also raise the risk of stroke, it does this by promoting blood-brain barrier disruption and cerebrovascular inflammation. People with ACS are more likely to have this if they also have high blood pressure in the lungs or problems with the right side of the heart. People with ACS often also have metabolic problems. These include high blood pressure, diabetes, and being overweight. These problems can make it harder for the kidneys to remove uric acid, especially by changing how a protein called URAT1 works. This leads to higher uric acid levels and creates a cycle that makes inflammation-related ACS get worse. The precise causal sequence of these interactions often hinges on underlying comorbidities and individualized pathophysiological profiles.

The findings of this study reveal that HSUA substantially elevates the risks of all-cause mortality, cardiovascular mortality, MACE, stroke, and heart failure among patients with ACS. These results align partially with those of a prior meta-analysis; however, the present investigation extends these insights by incorporating a more expansive evidence base (40 vs. 14 studies) and evaluating additional endpoints, such as stroke and heart failure, which were insufficiently addressed in earlier efforts. The nonlinear dose-response relationships for all-cause and cardiovascular mortality back up the idea of a threshold effect. Risks go up a lot when SUA levels go above a certain point. Because there is no link with revascularization, it seems that the severity of the coronary lesions and the choices made by doctors are the main factors that affect this outcome. The levels of SUA do not have a big effect on it. These ideas are backed up by subgroup analyses. They show that the effect is stronger in people with STEMI. This could mean that uric acid can hurt their hearts more easily.

Because of the considerable heterogeneity revealed in this meta-analysis, we further performed subgroup analyses to explore its sources. When HSUA group was compared with non-HSUA group, all-cause mortality, cardiovascular mortality, MACE (HR), and revascularization presented high heterogeneity; whereas stroke and heart failure presented moderate heterogeneity. Subgroup analyses indicated that follow-up time, ACS subtype, treatment method, and geographic region contributed to this high heterogeneity, and reduction of *I*^2^ could be observed in certain strata. For all-cause mortality, HSUA patients showed higher risk of acute events in short-term follow-up groups. Mid to long-term follow-up could decrease the risk differences of acute events in HSUA patients, which may weaken overall heterogeneity of all-cause mortality. This may be due to the fact that the nature of acute inflammation in ACS is amplified by HSUA-induced oxidative stress and NLRP3 inflammasome activity, and these effects could cause inconsistent early outcomes like faster myocardial ischemia or stroke (4, 5). Similarly, the large overall *I*^2^ of cardiovascular mortality may be correlated to disease subtype. For example, STEMI patients always present more severe ischemia-reperfusion injury and endothelial dysfunction, which may cause more overall *I*^2^ than cardiac acute events in NSTEMI patients with mostly chronic plaque instability (26, 44). In addition, the MACE heterogeneity seemed also related to ACS subtype distinctions. For STEMI/NSTEMI subgroup (HR = 1.40, 95% CI: 1.18–1.66; reduced *I*^2^), the amplified inflammatory response may increase composite risk in HSUA patients; whereas ACS broader groups also contributed to higher overall *I*^2^.Treatment method including PCI vs. non-PCI strategies also contributed substantially to this heterogeneity. Post-interventional inflammatory cascade may differentially modulate the effects of HSUA on vascular remodeling and neurological sequelae, causing discrepant outcome reporting between PCI and non-PCI strategies (35, 51, 56). In addition, geographic region also contributed, and Europe/Asia subgroups indicated different overall I², such as higher HR in Europe for all-cause mortality, 2.28, 95% CI: 1.53–3.40, which may be related to differences in population genetics, dietary urate intake, healthcare access, and comorbid profiles like hypertension and diabetes prevalence. In addition, emerging evidence, including findings from the URRAH project, suggests that lower SUA cut-off values (e.g., >5.1 mg/dL for females and >5.6 mg/dL for males) may more accurately predict cardiovascular events and mortality than traditional thresholds (60). Although diuretic use—a common contributor to hyperuricemia—was adjusted for or reported in the baseline characteristics of many included studies, residual confounding may still be present (61). This could partly explain the heterogeneity observed in our findings and highlights the need for further validation in ACS cohorts.

For stroke and heart failure, differences between studies were smaller. Subgroup analyses elucidated specific influences on these outcomes, particularly follow-up time, ACS subtype, treatment method, and geographic region, with several subgroups showing reduced heterogeneity. Stroke risk showed a HR of 1.45 in short-term follow-up groups and 2.11 in STEMI groups. This may be because HSUA patients have higher risk for blood vessel events during the acute phase. This is caused by differences in thrombogenesis or inflammatory processes. Long-term data may show reduced risk through inflammation control and active medical treatments. In heart failure, patients with HSUA showed consistently higher risk across multiple subgroups, with the association most pronounced in European cohorts. These factors include dietary patterns, lifestyle, or differences in medical intervention standards.

This study highlights SUA as a prognostic marker in ACS, showing its link to adverse outcomes. The heterogeneity observed suggests that HSUA's role in ACS prognosis is influenced by clinical, methodological, and demographic factors. It explains how uric acid contributes to cardiovascular issues through inflammation and oxidative stress. The risks associated with HSUA suggest the potential of urate-lowering therapies in ACS treatment. Future research should use standardized SUA thresholds, consider confounders like diuretic use, and employ larger, diverse prospective cohorts to refine these findings.

## 5 Conclusion

The clinical value of SUA as a possible target for intervention, HSUA greatly increases the risk of all-cause mortality, cardiovascular mortality, MACE, stroke, and heart failure in these patients and has a nonlinear mortality connection. However, other factors may confound these conclusions. There should be more high-quality randomized controlled trials to confirm the effect of controlling blood uric acid on prognosis.

## Data Availability

The original contributions presented in the study are included in the article/Supplementary Material, further inquiries can be directed to the corresponding authors.
