# Supplementary material for: Serum uric acid level and prognosis of acute coronary syndrome: a systematic review and dose-response meta-analysis
Source: Front Cardiovasc Med. 2026 Jan 9;12:1670418. doi: 10.3389/fcvm.2025.1670418 (PMC12827606; doi:10.3389/fcvm.2025.1670418)
Supplement: Supplementary file 1 [file Datasheet1.docx]

**Supplementary Material**

**Supplementary Table S1 Retrieval Strategy**

**Supplementary Table S2 of Baseline Characteristics**

**Supplementary Table S3 Newcastle–Ottawa Scale (NOS) scoring**

**Supplementary Table S4 Quality Evaluation**

**Supplementary Table S5 HSUA versus non-HSUA**

**Supplementary Table S6 PRISMA 2020 Checklist**

**Supplementary Figure S1 Subgroup analysis images.**

**Supplementary Figure S2 Sensitivity and publication bias analysis.**

**Supplementary Table S1 Retrieval Strategy**

Web of science

| #1 | Uric Acid (Topic) OR Acid, Uric (Topic) OR Trioxopurine (Topic) OR Urate (Topic) and Preprint Citation Index (Exclud–Database) |
| --- | --- |
| #2 | Hyperuricemia (Topic) OR Hyperurica* (Topic) and Preprint Citation Index (Exclude –Database) |
| #3 | #1 OR #2 and Preprint Citation Index (Exclude – Database) |
| #4 | Angina, Unstable (Topic) OR Anginas, Unstable (Topic) OR Unstable Anginas (Topic) OR Angina at Rest (Topic) OR Preinfarction Angina (Topic) OR Preinfarction Anginas (Topic) OR Unstable Angina (Topic) OR Unstable Angina Pectori (Topic) OR Myocardial Preinfarction Syndrome (Topic) OR Myocardial Preinfarction Syndromes (Topic) OR unstable angina pectoris (Topic) and Preprint Citation Index (Exclude – Database) |
| #5 | Myocardial Infarction (Topic) OR Myocardial Infarctions (Topic) OR Heart Attack (Topic) OR Heart Attacks (Topic) OR Myocardial Infarct (Topic) OR Myocardial Infarcts (Topic) OR Cardiovascular Stroke (Topic) OR Cardiovascular Strokes (Topic) OR heart infarction (Topic) OR cardiac infarct (Topic) OR cardiac infarction (Topic) OR cardial infarct (Topic) OR heart attack (Topic) OR heart infarct (Topic) OR myocardial infarct (Topic) OR myocardial infarction (Topic) OR myocardium infarct (Topic) OR myocardium infarction (Topic) OR premonitory infarction sign (Topic) OR subendocardial infarction (Topic) and Preprint Citation Index (Exclude – Database) |
| #6 | Acute Coronary Syndrome (Topic) OR Acute Coronary Syndromes (Topic) OR Coronary Syndrome, Acute (Topic) OR Coronary Syndromes, Acute (Topic) OR Syndrome, Acute Coronary (Topic) OR Syndromes, Acute Coronary (Topic) and Preprint Citation Index (Exclude – Database) |
| #7 | #6 OR #5 OR #4 and Preprint Citation Index (Exclude – Database) |
| #8 | #7 AND #3 and Preprint Citation Index (Exclude – Database) |

PubMed

| #1 | "Uric Acid"[MeSH Terms] |
| --- | --- |
| #2 | (((Uric Acid[Title/Abstract]) OR (Acid, Uric[Title/Abstract])) OR (Trioxopurine[Title/Abstract])) OR (Urate[Title/Abstract]) |
| #3 | #1 OR #2 |
| #4 | "Hyperuricemia"[Mesh] |
| #5 | (Hyperuricemia[Title/Abstract]) OR (Hyperurica*[Title/Abstract]) |
| #6 | #4 OR #5 |
| #7 | #3 OR #6 |
| #8 | "Angina, Unstable"[Mesh] |
| #9 | ((((((((((Angina, Unstable[Title/Abstract]) OR (Anginas, Unstable[Title/Abstract])) OR (Unstable Anginas[Title/Abstract])) OR (Angina at Rest[Title/Abstract])) OR (Preinfarction Angina[Title/Abstract])) OR (Preinfarction Anginas[Title/Abstract])) OR (Unstable Angina[Title/Abstract])) OR (Unstable Angina Pectori[Title/Abstract])) OR (Myocardial Preinfarction Syndrome[Title/Abstract])) OR (Myocardial Preinfarction Syndromes[Title/Abstract])) OR (unstable angina pectoris[Title/Abstract]) |
| #10 | #8 OR #9 |
| #11 | "Myocardial Infarction"[Mesh] |
| #12 | (((((((((((((((((Myocardial Infarction[Title/Abstract]) OR (Myocardial Infarctions[Title/Abstract])) OR (Heart Attack[Title/Abstract])) OR (Heart Attacks[Title/Abstract])) OR (Myocardial Infarct[Title/Abstract])) OR (Myocardial Infarcts[Title/Abstract])) OR (Cardiovascular Stroke[Title/Abstract])) OR (Cardiovascular Strokes[Title/Abstract])) OR (heart infarction[Title/Abstract])) OR (cardiac infarct[Title/Abstract])) OR (cardiac infarction[Title/Abstract])) OR (cardial infarct[Title/Abstract])) OR (heart infarct[Title/Abstract])) OR (myocardial infarction[Title/Abstract])) OR (myocardium infarct[Title/Abstract])) OR (myocardium infarction[Title/Abstract])) OR (premonitory infarction sign[Title/Abstract])) OR (subendocardial infarction[Title/Abstract]) |
| #13 | #11 OR #12 |
| #14 | "Acute Coronary Syndrome"[Mesh] |
| #15 | (((((Acute Coronary Syndrome[Title/Abstract]) OR (Acute Coronary Syndromes[Title/Abstract])) OR (Coronary Syndrome, Acute[Title/Abstract])) OR (Coronary Syndromes, Acute[Title/Abstract])) OR (Syndrome, Acute Coronary[Title/Abstract])) OR (Syndromes, Acute Coronary[Title/Abstract]) |
| #16 | #14 OR #15 |
| #17 | #10 OR #13 OR #16 |
| #18 | #7 AND #17 |

Embase

| #1 | 'uric acid'/exp |
| --- | --- |
| #2 | 'uric acid':ab,ti OR 'acid, uric':ab,ti OR trioxopurine:ab,ti OR urate:ab,ti |
| #3 | #1 OR #2 |
| #4 | 'hyperuricemia'/exp |
| #5 | hyperuricemia:ab,ti OR hyperurica*:ab,ti |
| #6 | #4 OR #5 |
| #7 | #3 OR #6 |
| #8 | 'unstable angina pectoris'/exp |
| #9 | 'unstable angina pectoris':ab,ti OR 'angina, unstable':ab,ti OR 'anginas, unstable':ab,ti OR 'unstable anginas':ab,ti OR 'angina at rest':ab,ti OR 'preinfarction angina':ab,ti OR 'preinfarction anginas':ab,ti OR 'unstable angina':ab,ti OR 'unstable angina pectori':ab,ti OR 'myocardial preinfarction syndrome':ab,ti OR 'myocardial preinfarction syndromes':ab,ti |
| #10 | #8 OR #9 |
| #11 | 'heart infarction'/exp |
| #12 | 'heart infarction':ab,ti OR 'myocardial infarctions':ab,ti OR 'heart attack':ab,ti OR 'heart attacks':ab,ti OR 'myocardial infarct':ab,ti OR 'myocardial infarcts':ab,ti OR 'cardiovascular stroke':ab,ti OR 'cardiovascular strokes':ab,ti OR 'cardiac infarct':ab,ti OR 'cardiac infarction':ab,ti OR 'cardial infarct':ab,ti OR 'heart infarct':ab,ti OR 'myocardial infarction':ab,ti OR 'myocardium infarct':ab,ti OR 'myocardium infarction':ab,ti OR 'premonitory infarction sign':ab,ti OR 'subendocardial infarction':ab,ti |
| #13 | #11 OR #12 |
| #14 | 'acute coronary syndrome'/exp |
| #15 | 'acute coronary syndrome':ab,ti OR 'acute coronary syndromes':ab,ti OR 'coronary syndrome, acute':ab,ti OR 'coronary syndromes, acute':ab,ti OR 'syndrome, acute coronary':ab,ti OR 'syndromes, acute coronary':ab,ti |
| #16 | #14 OR #15 |
| #17 | #10 OR #13 OR #16 |
| #18 | #7 AND #17 |

Cochrane

| #1 | MeSH descriptor: [Uric Acid] explode all trees |
| --- | --- |
| #2 | (Uric Acid OR Acid, Uric OR Trioxopurine OR Urate):ti,ab,kw |
| #3 | #1 or #2 |
| #4 | MeSH descriptor: [Hyperuricemia] explode all trees |
| #5 | (Hyperuricemia OR Hyperurica*):ti,ab,kw |
| #6 | #4 or #5 |
| #7 | #3 OR #6 |
| #8 | MeSH descriptor: [Angina, Unstable] explode all trees |
| #9 | (unstable angina pectoris OR Angina, Unstable OR Anginas, Unstable OR Unstable Anginas OR Angina at Rest OR Preinfarction Angina OR Preinfarction Anginas OR Unstable Angina OR Unstable Angina Pectori OR Myocardial Preinfarction Syndrome OR Myocardial Preinfarction Syndromes):ti,ab,kw |
| #10 | #8 OR #9 |
| #11 | MeSH descriptor: [Myocardial Infarction] explode all trees |
| #12 | (Myocardial Infarction OR Myocardial Infarctions OR Heart Attack OR Heart Attacks OR Myocardial Infarct OR Myocardial Infarcts OR Cardiovascular Stroke OR Cardiovascular Strokes OR heart infarction OR cardiac infarct OR cardiac infarction OR cardial infarct OR heart infarct OR myocardial infarction OR myocardium infarct OR myocardium infarction OR premonitory infarction sign OR subendocardial infarction):ti,ab,kw |
| #13 | #11 OR #12 |
| #14 | MeSH descriptor: [Acute Coronary Syndrome] explode all trees |
| #15 | (Acute Coronary Syndrome OR Acute Coronary Syndromes OR Coronary Syndrome, Acute OR Coronary Syndromes, Acute OR Syndrome, Acute Coronary OR Syndromes, Acute Coronary):ti,ab,kw |
| #16 | #14 OR #15 |
| #17 | #10 OR #13 OR #16 |
| #18 | #7 AND #17 |

**Supplementary Table S2 of Baseline Characteristics**

| **No** | **Author** | **Publication year** | **Data analysis methods** | **Fowllow-up time** | **Classification of SUA(mg/dl)** | **MACE definitions** |
| --- | --- | --- | --- | --- | --- | --- |
| 1 | Akpek et al. | 2011 | Age, Killip’s classification, peak creatine phosphokinase level | In-hospital period | ≥5.4/<5.4 | in stent thrombosis, non-fatal myocardial infarction and in-hospital mor tality during in-hospital follow up period. |
| 2 | Basar et al. | 2011 | Age, Mean platelet volume,C-reactive protein, sexual status, smoking, glucose on admission, multivessel disease | 1 year | >6.5/≤5.4 | - |
| 3 | Chen et al. | 2012 | TIMI risk score,Prior MI,ST segment elevation resolution,Abnormal TIMI myocardial per fusion grade,corrected TIMI frame count,left ventricular ejection fraction,Multivessel disease,Peak creatine kinase MB,Peak troponin I,high-sensitivity C-reactive protein,Creatinine,left ventricular ejection fraction | In-hospital period | ≥ 6/<6 in women / ≥ 7/<7 in men | - |
| 4 | Kaya et al. | 2012 | - | In-hospital period/24.3 months/5 years | ＞ 6/≤6 in women / ＞ 7/≤7 in men | cardiovascular mortality, reinfarction, repeat target vessel revascularization (percutaneous or surgical), or acute stent thrombosis. |
| 5 | Krishnan et al. | 2012 | Cardiovascular Mortality: male gender, hypertension, smoking, previous bypasshistory, glomerular filtration rate 60 ml/min/1.73 m, time to reperfusion, mean platelet volume, stent length, left ventricular ejection fraction, creatine kinase-MB, admission anemia, anterior myocardial infarction; MACE: male gender, hypertension, smoking, previous bypasshistory, glomerular filtration rate 60 ml/min/1.73 m2, TIMI flow grade 3, time to reperfusion, mean platelet volume, stent length, left ventricular ejection fraction, creatine kinase-MB, anterior myocardial infarction. | average of 2.8 years | 2.38-4.48/4.5-5.42/5.47-6.48/6.52-11.05 | - |
| 6 | Ndrepepa et al. | 2012 | Age, gender, African American ethnicity, body mass index, blood pressure/hypertension, smoking, diabetes status, physical activity measure, hyperlipidemia | 1 year | 1.3-5.3/5.3-6.3/6.3-7.5/7.5-18.4 | - |
| 7 | Omidvar et al. | 2012 | - | In-hospital period | >7 / ≤7 | - |
| 8 | Wang et al. | 2012 | Age, hypertension, Diabetes Mellitus, dyslipidemia, smoking, Body Mass Index, ejection fraction, serum creatinine level | In-hospital period | ≥5.8 / <5.8 | - |
| 9 | Levantesi et al. | 2013 | - | 3.5 years | ≤4.5 / 4.6-5.3/ 5.4-6.0 / 6.1-6.8 />6.8 | - |
| 10 | Timóteo et al. | 2013 | Age, gender, history of diabetes, history of hypertension, total cholesterol, body mass index, left ventricular ejection fraction, claudication intermittens, residual ischemia, electrical instability, glomerular filtration rate, dietary score, smoking, n−3 PUFA, vitamin-E, antiplatelet agents, angiotensin-converting-enzyme inhibitor, lipid-lowering medication, beta-blockers, and diuretics | 1 year | ≥6.25 / <6.25 | - |
| 11 | Akgul et al. | 2014 | Age, male gender, diabetes, heart rate,systolic blood pressure, angiotensin-converting enzyme inibitorsand beta-blocker use, Killip class ≥ 2, estimated glomerular filtration rate < 60 ml/min/1.73 m²,percutaneous coronary intervention | 6 months | >5.7 / ≤5.7 | - |
| 12 | Gazi et al. | 2014 | Age、Three-vessel disease、Unsuccessful procedure、left ventricular ejection fraction<40% | In-hospital period | ＞ 6/≤6 in women / ＞ 7/≤7 in men | - |
| 13 | Karim et al. | 2015 | Age ≥70 years, no thrombolytic treatment, renal failure, female gender, chest pain time >6 hours, No angiotensin-converting enzyme inhibitor treatment，No beta-blocker treatment | In-hospital period | - | complication which occurs after acute coronary syndrome such as cardiogenic syock, acute heart failure, stroke, reinfarct during early hospitalization, sudden cardiac death, repeat PCI during hospitalization, and coronary artery bypass graft (CABG) surgery. |
| 14 | Lazzeri et al. | 2015 | Renal function,Dyslipidemia,Hypertension,Diabetes mellitus | 1 year | ≤ 4.7 / 4.8–6.0 /≥6.1 | - |
| 15 | Von Lueder et al. | 2015 | - | 3 years | 0.8- 4.7 / 4.7- 5.8 / 5.8-7.1/ 7.1- 27.6 | - |
| 16 | Hajizadeh et al. | 2016 | Age, gender, Killip class (III-IV vs. I-II), systolic blood pressure, co-morbidities (diabetes, hypertension, renal insufficiency,chronic obstructive pulmonary disease, and peripheral arterydisease), medication use (beta-blockers, angiotensin-converting enzyme inibitors and/or angiotensin receptor blocker, and diuretics),body weight >85 kg and biological variables (haemoglobin, natraemia, and estimated glomerular filtration rate <60 mL/min/1.73 m2). | 20 months | ＞ 7.5/≤7.5 in women / ＞ 8/≤8 in men | - |
| 17 | Ranjith et al. | 2016 | - | In-hospital period | ≥ 7.2/<7.2 | - |
| 18 | Liu et al. | 2017 | Age ≥65 years, cerebrovascular accident, cardiogenic shock, ventricular arrhythmias, cardiac failure, complete heart block, recurrent myocardial infarction, high serum creatinine, atrial fibrillation, hyperuricemia, Killip classification ≥2, recurrence of angina, hypertension, diabetes, anemia, male sex, and heart rate >100 bpm | 30 days / 1 year | ＞ 6/≤6 in women / ＞ 7/≤7 in men | - |
| 19 | Magnoni et al. | 2017 | Age,male,body mass index, creatinine,intra-aortic balloon pump use,Killip's classification,Stent implantation | In-hospital period | >6.0 / ≤6.0 | - |
| 20 | Morn et al. | 2017 | Age, gender, estimated glomerular filtration rate, metabolic syndrome, revascularization, ethnicity | In-hospital period | ≥ 6/<6 in women / ≥ 7/<7 in men | - |
| 21 | Pagidipati et al. | 2017 | Age,sex,body mass index,hypertension,diabetes,killip class ≥ 2,previous acute coronary syndrome,Thrombolysis,Primary percutaneous coronary intervention | The median follow-up period of 365 days | ≥6 / <6 | - |
| 22 | Kobayashi et al. | 2018 | - | 2 years | ≤6.0 /6.1–7.0/7.1–8.0 / >8.0;＞8.0/≤8.0 | - |
| 23 | Kobayashi et al. | 2018 | Age，male gender,Diabetes mellitus,Hypertension,Dyslipidemia,Prior myocardial infarction,Creatinine, | 2 years | ≥6 / <6 | - |
| 24 | Tscharre et al. | 2018 | - | The median follow-up period of 5.5 ± 2.9 years | ≥ 6/<6 in women / ≥ 7/<7 in men | (1) cardiovascular death, (2) non-fatal myocardial infarction, and (3) non-fatal ischemic stroke. |
| 25 | Lopez et al. | 2018 | Age, gender, body-mass index, cardiogenic shock, TIMI minor or major bleeding, traditional cardiovascular risk factors, prior MI, prior coronary revascularisation (either percutaneous coronary intervention or coronary artery bypass graft), prior stroke or TIA, peripheral artery disease, atrial fibrillation, heart failure, malignancy, estimated glomerular filtra tion rate, peak troponin I, peak hs-C-reactive protein, serum blood glucose at admission, haematocrit at admission, vascular access site, number of affected coronary vessels, type of used stents, number of used stents, total stent length, and drug therapy (beta-blocker, angiotensin-converting enzyme inibitors or angiotensin receptor blocker, PPI, diuretics, high-intensity statins, allopurinol and antithrombotic-therapy) | 5 years | ＞5.7/≤5.7 in women / ＞7/≤7 in men | non-fatal ACS, unplanned revascularization, or readmission for any cardiovascular disease including heart failure, stroke or unstable angina. |
| 26 | Ye et al. | 2018 | Age, sex, cardiovascular risk factors (body mass index, hypertension, smoking habit, diabetes and dyslipidemia), glomerular filtration rate, previous coronary heart disease,heart failure or stroke, as well as medical treatments at discharge (clopidogrel, prasugrel, ticagrelor, dual antiplatelet treatment, betablockers, angiotensin-converting enzyme inibitors/angiotensin receptor blocker, statins, diuretics,espironolactone/eplerenone, nitrates, oral antidiabetics). | The median follow-up period of 246.31 ±49.16 days | ＞5.6 / <5.6 | - |
| 27 | Tai et al. | 2019 | Serum creatinine, triglyceride, and history of heart failure. | In-hospital period / 1 year | ＞ 6/≤6 in women / ＞7/≤7 in men | - |
| 28 | Guo et al. | 2019 | Age, body mass index, current smoking, percutaneous coronary intervention or coronary artery bypass graft history, hypertension, diabetes mellitus, chronic kidney disease ≥3, and stroke | The median follow-up of 2.3±1.0 years | ＞ 6/≤6 in women / ＞7/≤7 in men | - |
| 29 | Mandurino et al. | 2020 | Age ≥75,female,left ventricular ejection fraction≥40,chronic  kidney disease,hypotension,beta-blocker,angiotensin-converting enzyme  inhibitors / angiotensin receptor blocker | 1 year | ≥6.8 / < 6.8 | - |
| 30 | Centola et al. | 2020 | - | In-hospital period | ≥6.3 / < 6.3 | - |
| 31 | Ma et al. | 2021 | Male gender, age, previous AMI, previous PCI, previous surgical coronary revascularization, diabetes mellitus, hypertension, diagnosis of STEMI, multivessel coronary disease, renal impairment, anemia, GRACE score | The median follow-up of 41.7 months | ≥ 6/<6 in women / ≥ 7/<7 in men | - |
| 32 | Mohammed et al. | 2021 | Age, sex, body mass index，MI type, hypertension, diabetes and dyslipidemia | 30 months | ＞ 6/≤6 in women / ＞7/≤7 in men | - |
| 33 | Kim et al. | 2022 | Age, hypertension, diabetes, left ventricular ejection fraction, diuretic use,chronic kidney disease | The median follow-up of 5.02 (3.07, 7.55) years | ＞ 5.8/≤5.8 in women / ＞6.5/≤6.5 in men | - |
| 34 | Nakahashi et al. | 2022 | Age,sex,body mass index,hypertension,diabetes mellitus,dyslipidemia,smoking status,chronic kidney disease,left ventricular ejection fraction,estimated glomerular filtration rate | In-hospital period | ≥ 6/<6 in women / ≥ 7/<7 in men | - |
| 35 | Tang et al. | 2022 | Age,sex, diabetes mellitus,estimated glomerular filtration rate <60 mL/min/1.73 m2, high-sensitivity C-reactive protein＞0.15mg/dl,multivessel disease,Suboptimal coronary flow after percutaneous coronary intervention,cardiogenic shock,anemia | 5 years | ＞ 6/≤6 in women / ＞ 7/≤7 in men | all-cause death, MI, unplanned revascularization, or stroke |
| 36 | Nakahashi et al. | 2022 | Age, gender, hypertension, hyperlipidemia, diabetes mellitus, chronic kidney disease, current smoker, family history of coronary heart disease, left ventricular ejection fraction, SYNTAX score, old myocardial infarction, previous percutaneous coronary intervention, previous coronary artery bypass graft , and inflammatory markers. | The median of 4 years | ≤4.4 / 4.5-7.1 / ≥7.2 | - |
| 37 | Dyrbuś et al. | 2023 | Age, sex, hypertension, diabetes mellitus, dyslipidemia, smoking status, renal function (assessed by estimated glomerular filtration rate or serum creatinine), high-sensitivity C-reactive protein, hemoglobin, left ventricular ejection fraction, Killip classification, and the presence of multivessel coronary artery disease. | 3 years | ＜4.9/4.9-5.9/5.9-7.2/＞7.2 | - |
| 38 | Liang et al. | 2023 | - | 1 year | ＞ 6/≤6 in women /＞7/≤7 in men | - |
| 39 | Nie et al. | 2024 | Age, sex, cardiovascular risk factors (hypertension, diabetes, current smoker, prior MI, and prior stroke), PPCI, Killip class at admission, estimated glomerular filtration rate, glucose, high-density lipoprotein cholestero, triglyceride, low-density lipoprotein cholesterol, medical treatments at discharge (Statins, angiotensin-converting enzyme inibitors/angiotensin receptor blocker, Beta-blocker, Diuretics) andleft ventricular ejection fraction | 1 year | >7 / ≤7 | - |
| 40 | Li et al. | 2025 |  | The median follow-up of 64 (46, 79) months | >7 / ≤7 | non-fatal myocardial infarction, non-fatal stroke/transient ischemic attack (TIA), unplanned target vessel revascularization, readmission due to heart failure, and all-cause mortality. |

**Supplementary Table S3:Newcastle–Ottawa Scale (NOS) scoring**

Newcastle-Ottawa quality assessment scale specific for this meta-analysis.

| **Selection:** (Maximum 4 stars) |
| --- |
| 1)Representativeness of the sample: |
| a) Truly representative of the average in the target population. * (all subjects or random sampling) |
| b) Somewhat representative of the average in the target population. (non- random sampling) |
| c) Selected group of users. |
| d)No description of the sampling strategy. |
| 2) Sample size: |
| a) Justified and satisfactory. * |
| b) Not justified. |
| 3) Ascertainment of the risk factor (uric acid): |
| a) Validated measurement tool(e.g., SUA measured via standardized laboratory assays such as enzymatic colorimetric methods, uricase-peroxidase, or high-performance liquid chromatography, with clear description in the study methods and quality control mentioned). ** |
| b) Non-validated measurement tool, but the tool is available or described(e.g., SUA measured via routine clinical lab tests without full validation details, but the method is explicitly stated, such as "serum uric acid levels were determined using an automated analyzer"). * |
| c) No description of the measurement tool or method. |
| **Comparability:** (Maximum 2 stars) |
| 1) The subjects in different groups are comparable, based on the study design or analysis. Confounding factors are controlled. |
| 1. Study controls for minimum dataset: age, sex, and one cardiovascular risk factor (e.g., hypertension, diabetes, or eGFR, relevant to SUA and ACS prognosis). * |
| 1. Controls for minimum dataset plus additional factors (e.g., smoking, BMI, lipids, ACS type, or treatment like PCI). ** |
| c) No control or univariate analysis only. |
| **Outcome:** (Maximum 3 stars) |
| 1) Assessment of the outcome : |
| a) Independent blind assessment or record linkage (e.g., outcomes from hospital records, national death registries, or electronic health records with verification). * |
| b) Self-report, structured interview, or follow-up visits/telephone without blind assessment (e.g., patient-reported events confirmed by charts). * |
| c) No description. |
| 1. Was follow-up long enough for outcomes to occur? |
| a) Follow-up ≥6 months for middle/long-term ACS outcomes (e.g., mortality, MACE, stroke, heart failure; sufficient per GRACE score timelines). * |
| b) Follow-up <6 months or not described. |
| 1. Adequacy of statistical test |
| a)Thestatisticaltestusedtoanalyzethedataisclearlydescribedandappropriate,andthemeasurement of the association is presented, including confidence intervals and the probability level(pvalue) (e.g., multivariable Cox regression for HR or logistic regression for OR).* |
| b)Thestatisticaltestisnotappropriate,notdescribedorincomplete |

**Supplementary Table S4 Quality Evaluation**

| **Study** | **Selection** | | | | **Comparability** | **Outcome** | | | **Quality scores** |
| --- | --- | --- | --- | --- | --- | --- | --- | --- | --- |
|  | Representativeness of the sample: | Sample size: | Ascertainment of the risk factor (uric acid): | Demonstration that outcome of interest was not present at start of study | The subjects in different groups are comparable, based on the study design or analysis. Confounding factors are controlled. | Assessment of the outcome : | Was follow-up long enough for outcomes to occur? | Adequacy of statistical test |  |
| Akpek2011 | **⭐** | **⭐** | **⭐** | **⭐** | **⭐⭐** | **⭐** | **⭐** | **-** | 8 |
| Basar 2011 | **-** | **⭐** | **-** | **⭐** | **⭐⭐** | **⭐** | **⭐** | **-** | 6 |
| Chen2012 | **⭐** | **⭐** | **⭐** | **⭐** | **-** | **⭐** | **⭐** | **⭐** | 7 |
| Kaya2012 | **⭐** | **⭐** | **-** | **⭐** | **⭐⭐** | **⭐** | **-** | **-** | 6 |
| Krishnan2012 | **⭐** | **⭐** | **-** | **-** | **⭐** | **⭐** | **⭐** | **⭐** | 6 |
| Ndrepepa2012 | **⭐** | **⭐** | **⭐** | **⭐** | **⭐⭐** | **⭐** | **⭐** | **-** | 8 |
| Omidvar2012 | **⭐** | **⭐** | **⭐** | **⭐** | **⭐⭐** | **-** | **⭐** | **-** | 7 |
| Wang2012 | **⭐** | **⭐** | **⭐** | **⭐** | **⭐⭐** | **⭐** | **⭐** | **-** | 8 |
| Levantesi2024 | **⭐** | **⭐** | **⭐** | **⭐** | **⭐⭐** | **⭐** | **⭐** | **⭐** | 9 |
| Timóteo2013 | **⭐** | **⭐** | **⭐** | **⭐** | **⭐⭐** | **⭐** | **⭐** | **⭐** | 9 |
| Akgul2014 | **⭐** | **⭐** | **-** | **-** | **⭐⭐** | **⭐** | **⭐** | **-** | 6 |
| Gazi2014 | **⭐** | **⭐** | **⭐** | **⭐** | **⭐⭐** | **⭐** | **⭐** | **-** | 8 |
| Karim2015 | **⭐** | **⭐** | **⭐** | **⭐** | **⭐** | **⭐** | **⭐** | **⭐** | 8 |
| Lazzeri2015 | **⭐** | **⭐** | **⭐** | **⭐** | **-** | **⭐** | **⭐** | **⭐** | 7 |
| Von Lueder2015 | **⭐** | **⭐** | **⭐** | **⭐** | **-** | **⭐** | **⭐** | **⭐** | 7 |
| Hajizadeh2016 | **⭐** | **-** | **⭐** | **⭐** | **⭐** | **⭐** | **⭐** | **-** | 6 |
| Ranjith2016 | **-** | **-** | **⭐** | **⭐** | **⭐⭐** | **⭐** | **⭐** | **-** | 6 |
| Liu2017 | **⭐** | **⭐** | **⭐** | **⭐** | **⭐⭐** | **⭐** | **⭐** | **⭐** | 9 |
| Magnoni2017 | **⭐** | **⭐** | **⭐** | **⭐** | **⭐⭐** | **⭐** | **⭐** | **-** | 8 |
| Mora2017 | **⭐** | **-** | **⭐** | **-** | **⭐⭐** | **⭐** | **⭐** | **-** | 6 |
| Pagidipati2017 | **⭐** | **⭐** | **⭐** | **⭐** | **⭐⭐** | **⭐** | **⭐** | **-** | 8 |
| Kobayashi2018 | **⭐** | **⭐** | **⭐** | **⭐** | **⭐⭐** | **⭐** | **⭐** | **-** | 8 |
| Kobayashi2018 | **⭐** | **⭐** | **⭐** | **⭐** | **⭐** | **⭐** | **⭐** | **⭐** | 8 |
| Tscharre2018 | **⭐** | **⭐** | **⭐** | **⭐** | **⭐⭐** | **⭐** | **⭐** | **⭐** | 9 |
| Lopez 2018 | **⭐** | **⭐** | **⭐** | **⭐** | **⭐⭐** | **⭐** | **⭐** | **-** | 8 |
| Ye2018 | **⭐** | **⭐** | **⭐** | **⭐** | **⭐⭐** | **⭐** | **⭐** | **-** | 8 |
| Tai2019 | **⭐** | **⭐** | **⭐** | **-** | **⭐** | **⭐** | **⭐** | **-** | 6 |
| Guo2019 | **⭐** | **⭐** | **⭐** | **⭐** | **⭐⭐** | **⭐** | **⭐** | **-** | 8 |
| Mandurino2020 | **⭐** | **⭐** | **⭐** | **-** | **⭐** | **⭐** | **⭐** | **-** | 6 |
| Centola2020 | **⭐** | **⭐** | **⭐** | **⭐** | **⭐⭐** | **⭐** | **⭐** | **⭐** | 9 |
| Ma2021 | **⭐** | **⭐** | **⭐** | **⭐** | **⭐⭐** | **⭐** | **⭐** | **⭐** | 9 |
| Mohammed2021 | **⭐** | **⭐** | **⭐** | **⭐** | **⭐** | **⭐** | **⭐** | **⭐** | 8 |
| Kim2022 | **⭐** | **-** | **⭐** | **⭐** | **⭐** | **⭐** | **⭐** | **⭐** | 7 |
| Nakahashi2022 | **⭐** | **-** | **⭐** | **-** | **⭐** | **⭐** | **⭐** | **⭐** | 6 |
| Tang2022 | **⭐** | **⭐** | **⭐** | **⭐** | **⭐⭐** | **⭐** | **⭐** | **-** | 8 |
| Nakahashi2022 | **⭐** | **⭐** | **⭐** | **⭐** | **⭐⭐** | **⭐** | **⭐** | **-** | 8 |
| Dyrbuś2023 | **⭐** | **⭐** | **⭐** | **-** | **⭐⭐** | **⭐** | **⭐** | **-** | 7 |
| Liang2023 | **-** | **⭐** | **⭐** | **-** | **⭐** | **⭐** | **⭐** | **⭐** | 6 |
| Nie2024 | **⭐** | **⭐** | **⭐** | **⭐** | **⭐⭐** | **⭐** | **⭐** | **-** | 8 |
| Li2025 | **⭐** | **⭐** | **⭐** | **⭐** | **⭐⭐** | **⭐** | **⭐** | **⭐** | 9 |

**Supplementary Table S5 HSUA versus non-HSUA**

| **Group** | **Number of studies** | **Heterogeneity I², P** | **HR, 95% CI** | **P** |
| --- | --- | --- | --- | --- |
| **All-Cause Mortality** |  |  |  |  |
| overall | 12 | 66.1%, p=0.001 | 1.81 (1.47-2.22) | P<0.001 |
| Follow-up time |  |  |  |  |
| Middle to long | 11 | 68.0%,p=0.001 | 1.80 (1.44-2.23) | P<0.001 |
| Short | 2 | 0.0%,p=0.434 | 1.69 (1.08-2.64) | p=0.022 |
| Type of ACS |  |  |  |  |
| STEMI | 6 | 25.8%,p=0.241 | 2.07 (1.59-2.70) | P<0.001 |
| ACS | 3 | 46.6%,p=0.154 | 1.84 (1.23-2.76) | p=0.003 |
| STEMI/NSTEMI | 3 | 63.2%,p=0.066 | 1.43 (1.08-1.89) | P=0.013 |
| Treatment methods |  |  |  |  |
| PCI | 6 | 71.7%,p=0.004 | 1.64 (1.24-2.17) | p=0.001 |
| PCI/non-PCI | 6 | 24.5%,p=0.250 | 1.95 (1.53-2.50) | P<0.001 |
| Region |  |  |  |  |
| Europe | 4 | 59.2%,p=0.061 | 2.28 (1.53-3.40) | P<0.001 |
| Asia | 7 | 51.7%,p=0.053 | 1.55 (1.25-1.92) | P<0.001 |
| America | 1 | - | 1.99 (1.08-3.66) | p=0.027 |
| **Group** | **Number of studies** | **Heterogeneity I², P** | **OR, 95% CI** | **P** |
| **All-Cause Mortality** |  |  |  |  |
| overall | 7 | 72.4%, p=0.001 | 1.97 (1.29-2.99) | P=0.002 |
| Follow-up time |  |  |  |  |
| Middle to long | 2 | 0%, p=0.629 | 1.17 (1.03-1.32) | p<0.05 |
| Short | 5 | 46.4%,p=0.113 | 2.67 (1.60-4.45) | p<0.001 |
| Type of ACS |  |  |  |  |
| STEMI | 2 | 61.6%, p=0.106 | 1.67 (0.58-4.86) | P=0.345 |
| ACS | 2 | 65.3%, p=0.090 | 4.69  (1.39-15.85) | p<0.05 |
| STEMI/NSTEMI | 3 | 0%,p=0.589 | 1.63 (1.17-2.26) | p<0.05 |
| Treatment methods |  |  |  |  |
| PCI | 2 | 63.2%,p=0.099 | 1.40 (0.82-2.40) | P=0.215 |
| PCI/non-PCI | 5 | 61.6%,p=0.034 | 2.43 (1.37-4.31) | p<0.05 |
| Region |  |  |  |  |
| Europe | 3 | 87.2%,p<0.001 | 2.73 (0.88-8.47) | p=0.081 |
| Asia | 3 | 13.9%,p=0.313 | 1.74(1.10-2.74) | p<0.05 |
| Africa | 1 | - | 1.70 (1.02-2.84) | p=0.043 |
| **Group** | **Number of studies** | **Heterogeneity I², P** | **HR, 95% CI** | **P** |
| **Mace** |  |  |  |  |
| overall | 7 | 82.1%,p<0.001 | 1.40 (1.15-1.71) | P=0.001 |
| Type of ACS |  |  |  |  |
| STEMI | 1 | - | 1,09(0.82-1.45) | P=0.001 |
| STEMI/NSTEMI | 2 | 0.0%,p=0.623 | 1.40 (1.18-1.66) | P<0.001 |
| ACS | 4 | 88.0%,p<0.001 | 1.55(1.08-2.22) | p=0.018 |
| Region |  |  |  |  |
| Others | 3 | 86.3%,p=0.001 | 1.29(1.00-1.67) | P=0.046 |
| Asia | 4 | 68.3%,p=0.024 | 1.54 (1.11-2.14) | p=0.009 |
| **Group** | **Number of studies** | **Heterogeneity I², P** | **OR, 95% CI** | **P** |
| **Mace** |  |  |  |  |
| overall | 3 | 36.5%,p=0.207 | 2.25 (1.73-2.92) | p<0.001 |
| Follow-up time |  |  |  |  |
| Middle to long | 2 | 0.0%,p=0.491 | 1.78 (1.21-2.61) | P=0.003 |
| Short | 2 | 0.0%,p=0.364 | 2.51 (1.86-3.39) | P<0.001 |
| **Group** | **Number of studies** | **Heterogeneity I², P** | **HR, 95% CI** | **P** |
| **Cardiovascular mortality** |  |  |  |  |
| overall | 9 | 92.9%,p<0.001 | 2.58 (1.67-3.98) | P<0.001 |
| Type of ACS |  |  |  |  |
| STEMI | 3 | 61.8%,p=0.073 | 3.84(2.34-6.32) | P<0.001 |
| ACS | 3 | 86.7%,p=0.001 | 2.73 (1.43-5.23) | p=0.002 |
| STEMI/NSTEMI | 3 | 79.2%,p=0.008 | 1.68 (1.11-2.53) | P<0.001 |
| Treatment methods |  |  |  |  |
| PCI | 3 | 97.6%,p<0.001 | 2.31 (0.84-6.29) | p= 0.103 |
| PCI/non-PCI | 6 | 75.8%,p=0.001 | 2.68(1.84-3.89) | P<0.001 |
| Region |  |  |  |  |
| Europe | 3 | 87.9%,p<0.001 | 3.50 (1.78-6.88) | P<0.001 |
| Asia | 5 | 91.0%,p<0.001 | 2.37 (1.33-4.24) | p=0.004 |
| America | 1 | - | 1.61 (1.16-2.23) | p=0.004 |
| **Group** | **Number of studies** | **Heterogeneity I², P** | **RR, 95% CI** | **P** |
| **Revascularization** |  |  |  |  |
| overall | 7 | 83.6%,p<0.001 | 1.09(0.80-1.47) | p = 0.594 |
| Follow-up time |  |  |  |  |
| Middle to long | 5 | 84.0%,p<0.001 | 1.26 (0.91-1.73) | p<0.001 |
| Short | 2 | 0.0%,p =0.662 | 0.67 (0.46-0.96) | p =0.662 |
| Type of ACS |  |  |  |  |
| STEMI | 3 | 83.6%,p =0.002 | 1.19 (0.70-2.02) | p =0.002 |
| ACS | 1 | - | 0.64 (0.43-0.96) | p<0.001 |
| STEMI/NSTEMI | 3 | 0.0%,p =0.728 | 1.08 (0.95-1.23) | p =0.728 |
| Treatment methods |  |  |  |  |
| PCI | 3 | 92.0%,p<0.001 | 1.23 (0.84-1.81) | p<0.001 |
| PCI/non-PCI | 4 | 32.0%,p =0.220 | 0.88 (0.58-1.33) | p =0.220 |
| Region |  |  |  |  |
| Europe | 2 | 69.3%,p =0.071 | 1.32 (0.63-2.79) | p =0.071 |
| Asia | 5 | 39.4%,p =0.159 | 0.97 (0.78-1.21) | p =0.159 |
| **Stroke** |  |  |  |  |
| overall | 12 | 47.2%,p=0.035 | 1.27(1.08-1.48) | p = 0.002 |
| Follow-up time |  |  |  |  |
| Middle to long | 7 | 39%,p=0.131 | 1.17 (0.96-1.41) | p = 0.117 |
| Short | 6 | 57.6%,p=0.038 | 1.45 (1.12-1.87) | p = 0.004 |
| Type of ACS |  |  |  |  |
| STEMI | 6 | 48.6%,p=0.084 | 2.11 (1.35-3.31) | p = 0.001 |
| ACS | 1 | - | 1.18 (0.95-1.46) | p = 0.138 |
| STEMI/NSTEMI | 5 | 48.4%,p=0.101 | 1.23 (0.96-1.58) | p = 0.108 |
| Treatment methods |  |  |  |  |
| PCI | 3 | 0.0%,p=0.452 | 1.26 (0.96-1.67) | p = 0.1 |
| PCI/non-PCI | 9 | 58.4%,p=0.014 | 1.27 (1.06-1.53) | p = 0.01 |
| Region |  |  |  |  |
| Europe | 2 | 3.4%,p=0.309 | 3.13 (1.28-7.63) | p = 0.012 |
| Asia | 7 | 57.3%,p=0.029 | 1.28 (1.00-1.63) | p = 0.052 |
| Others | 3 | 16.7%,p=0.301 | 1.21 (0.99-1.48) | p = 0.060 |
| **Heart failure** |  |  |  |  |
| overall | 10 | 44.8%,p=0.061 | 1.90 (1.72-2.11) | p = 0.000 |
| Follow-up time |  |  |  |  |
| Middle to long | 4 | 0.0%,p =0.432 | 1.95 (1.64-2.23) | p<0.001 |
| Short | 6 | 63.1%,p =0.019 | 1.88 (1.66-2.12) | p<0.001 |
| Type of ACS |  |  |  |  |
| STEMI | 5 | 0.0%,p =0.473 | 2.32(1.96- 2.74) | p<0.001 |
| STEMI/NSTEMI | 5 | 0.0%,p =0.440 | 1.71 (1.51-1.95) | p<0.001 |
| Treatment methods |  |  |  |  |
| PCI | 2 | 42.4%,p =0.187 | 1.90 (1.58-2.29) | p<0.001 |
| PCI/non-PCI | 8 | 51.9%,p =0.042 | 1.90(1.69- 2.14) | p<0.001 |
| Region |  |  |  |  |
| Europe | 3 | 0.0%,p =0.938 | 2.14 (1.75-2.63) | p<0.001 |
| Asia | 5 | 71.9%,p =0.007 | 1.89 (1.61-2.21) | p<0.001 |
| Others | 2 | 0.0%,p =0.061 | 1.76 (1.48-2.09) | p<0.001 |

**Supplementary Table S6. PRISMA 2020 Checklist**

| **Section and Topic** | **Item #** | **Checklist item** | **Location where item is reported** |
| --- | --- | --- | --- |
| **TITLE** | | |  |
| Title | 1 | Identify the report as a systematic review. | Page 1 |
| **ABSTRACT** | | |  |
| Abstract | 2 | See the PRISMA 2020 for Abstracts checklist. | Page 1 |
| **INTRODUCTION** | | |  |
| Rationale | 3 | Describe the rationale for the review in the context of existing knowledge. | Page 2 |
| Objectives | 4 | Provide an explicit statement of the objective(s) or question(s) the review addresses. | Page 2 |
| **METHODS** | | |  |
| Eligibility criteria | 5 | Specify the inclusion and exclusion criteria for the review and how studies were grouped for the syntheses. | Page 2-4 |
| Information sources | 6 | Specify all databases, registers, websites, organisations, reference lists and other sources searched or consulted to identify studies. Specify the date when each source was last searched or consulted. | Page 2-4 |
| Search strategy | 7 | Present the full search strategies for all databases, registers and websites, including any filters and limits used. | Page 2-4 |
| Selection process | 8 | Specify the methods used to decide whether a study met the inclusion criteria of the review, including how many reviewers screened each record and each report retrieved, whether they worked independently, and if applicable, details of automation tools used in the process. | Page 2-4 |
| Data collection process | 9 | Specify the methods used to collect data from reports, including how many reviewers collected data from each report, whether they worked independently, any processes for obtaining or confirming data from study investigators, and if applicable, details of automation tools used in the process. | Page 2-4 |
| Data items | 10a | List and define all outcomes for which data were sought. Specify whether all results that were compatible with each outcome domain in each study were sought (e.g. for all measures, time points, analyses), and if not, the methods used to decide which results to collect. | Page 2-4 |
|  | 10b | List and define all other variables for which data were sought (e.g. participant and intervention characteristics, funding sources). Describe any assumptions made about any missing or unclear information. | Page 2-4 |
| Study risk of bias assessment | 11 | Specify the methods used to assess risk of bias in the included studies, including details of the tool(s) used, how many reviewers assessed each study and whether they worked independently, and if applicable, details of automation tools used in the process. | Page 2-4 |
| Effect measures | 12 | Specify for each outcome the effect measure(s) (e.g. risk ratio, mean difference) used in the synthesis or presentation of results. | Page 2-4 |
| Synthesis methods | 13a | Describe the processes used to decide which studies were eligible for each synthesis (e.g. tabulating the study intervention characteristics and comparing against the planned groups for each synthesis (item #5)). | Page 2-4 |
|  | 13b | Describe any methods required to prepare the data for presentation or synthesis, such as handling of missing summary statistics, or data conversions. | Page 2-4 |
|  | 13c | Describe any methods used to tabulate or visually display results of individual studies and syntheses. | Page 2-4 |
|  | 13d | Describe any methods used to synthesize results and provide a rationale for the choice(s). If meta-analysis was performed, describe the model(s), method(s) to identify the presence and extent of statistical heterogeneity, and software package(s) used. | Page 2-4 |
|  | 13e | Describe any methods used to explore possible causes of heterogeneity among study results (e.g. subgroup analysis, meta-regression). | Page 2-4 |
|  | 13f | Describe any sensitivity analyses conducted to assess robustness of the synthesized results. | Page 2-4 |
| Reporting bias assessment | 14 | Describe any methods used to assess risk of bias due to missing results in a synthesis (arising from reporting biases). | Page 2-4 |
| Certainty assessment | 15 | Describe any methods used to assess certainty (or confidence) in the body of evidence for an outcome. | Page 2-4 |
| **RESULTS** | | |  |
| Study selection | 16a | Describe the results of the search and selection process, from the number of records identified in the search to the number of studies included in the review, ideally using a flow diagram. | Page 4-7 |
|  | 16b | Cite studies that might appear to meet the inclusion criteria, but which were excluded, and explain why they were excluded. | Page 4-7 |
| Study characteristics | 17 | Cite each included study and present its characteristics. | Page 4-7 |
| Risk of bias in studies | 18 | Present assessments of risk of bias for each included study. | Page 4-7 |
| Results of individual studies | 19 | For all outcomes, present, for each study: (a) summary statistics for each group (where appropriate) and (b) an effect estimate and its precision (e.g. confidence/credible interval), ideally using structured tables or plots. | Page 4-7 |
| Results of syntheses | 20a | For each synthesis, briefly summarise the characteristics and risk of bias among contributing studies. | Page 4-7 |
|  | 20b | Present results of all statistical syntheses conducted. If meta-analysis was done, present for each the summary estimate and its precision (e.g. confidence/credible interval) and measures of statistical heterogeneity. If comparing groups, describe the direction of the effect. | Page 4-7 |
|  | 20c | Present results of all investigations of possible causes of heterogeneity among study results. | Page 4-7 |
|  | 20d | Present results of all sensitivity analyses conducted to assess the robustness of the synthesized results. | Page 4-7 |
| Reporting biases | 21 | Present assessments of risk of bias due to missing results (arising from reporting biases) for each synthesis assessed. | Page 4-7 |
| Certainty of evidence | 22 | Present assessments of certainty (or confidence) in the body of evidence for each outcome assessed. | Page 4-7 |
| **DISCUSSION** | | |  |
| Discussion | 23a | Provide a general interpretation of the results in the context of other evidence. | Page 4-7 |
|  | 23b | Discuss any limitations of the evidence included in the review. | Page 4-7 |
|  | 23c | Discuss any limitations of the review processes used. | Page 4-7 |
|  | 23d | Discuss implications of the results for practice, policy, and future research. | Page 4-7 |
| **OTHER INFORMATION** | | |  |
| Registration and protocol | 24a | Provide registration information for the review, including register name and registration number, or state that the review was not registered. | Page 2-4 |
|  | 24b | Indicate where the review protocol can be accessed, or state that a protocol was not prepared. | Page 2-4 |
|  | 24c | Describe and explain any amendments to information provided at registration or in the protocol. | Page 2-4 |
| Support | 25 | Describe sources of financial or non-financial support for the review, and the role of the funders or sponsors in the review. | Page 8-9 |
| Competing interests | 26 | Declare any competing interests of review authors. | Page 8 |
| Availability of data, code and other materials | 27 | Report which of the following are publicly available and where they can be found: template data collection forms; data extracted from included studies; data used for all analyses; analytic code; any other materials used in the review. | Page 9 |

*From:*  Page MJ, McKenzie JE, Bossuyt PM, Boutron I, Hoffmann TC, Mulrow CD, et al. The PRISMA 2020 statement: an updated guideline for reporting systematic reviews. BMJ 2021;372:n71. doi: 10.1136/

**Supplementary Figure S1 Subgroup analysis images**

| Subgroup analysis of All-Cause Mortality(HR) | | |
| --- | --- | --- |
| **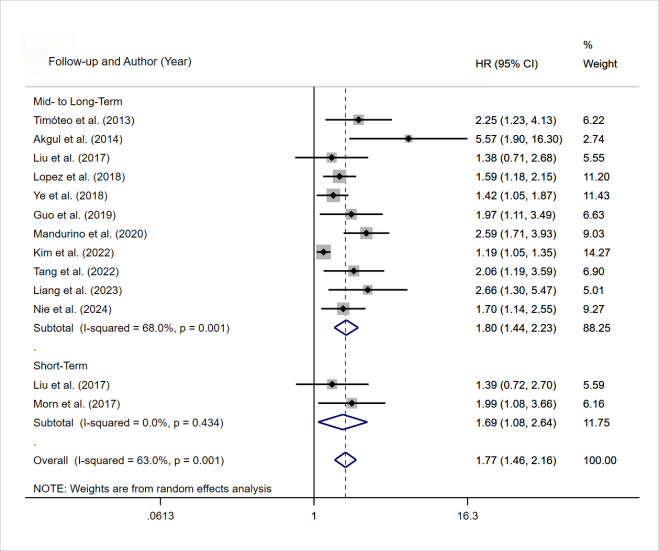** | | **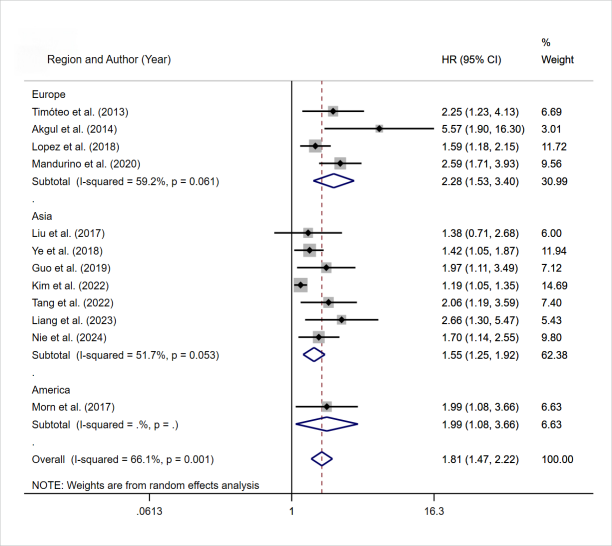** |
| **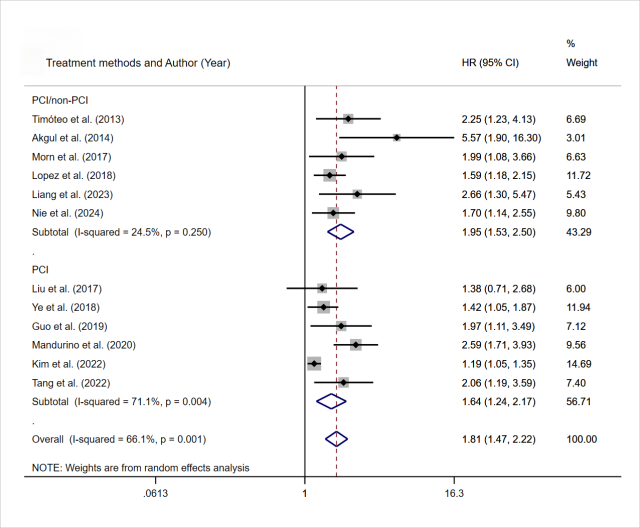** | | **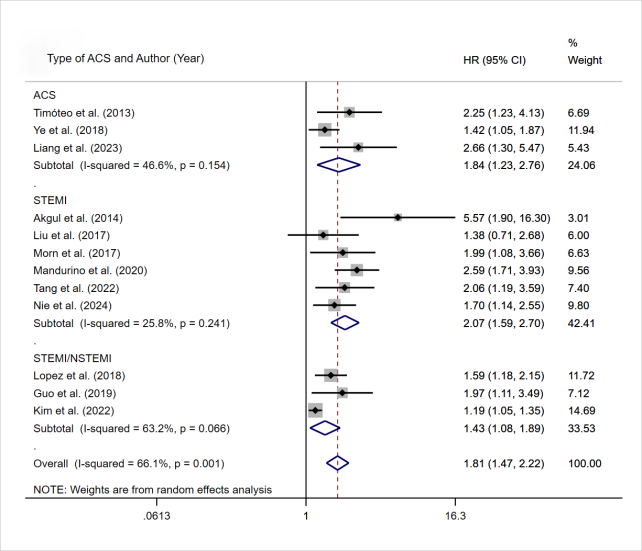** |
| Subgroup analysis of All-Cause Mortality(OR) | | |
| **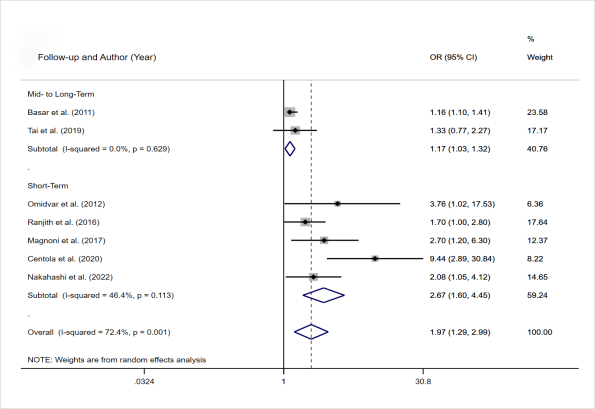** | | **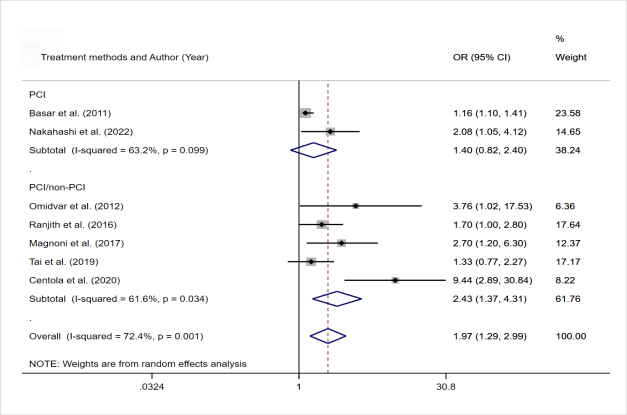** |
| **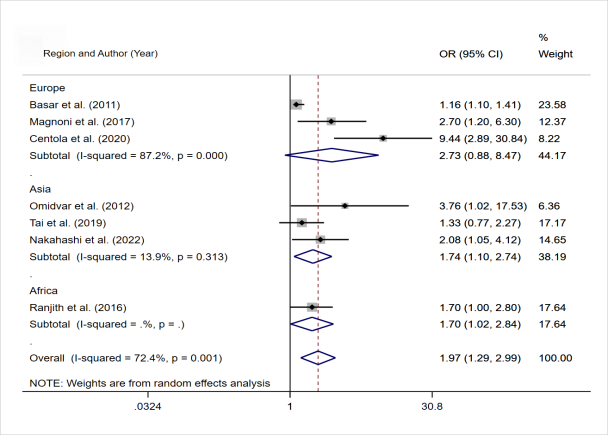** | | **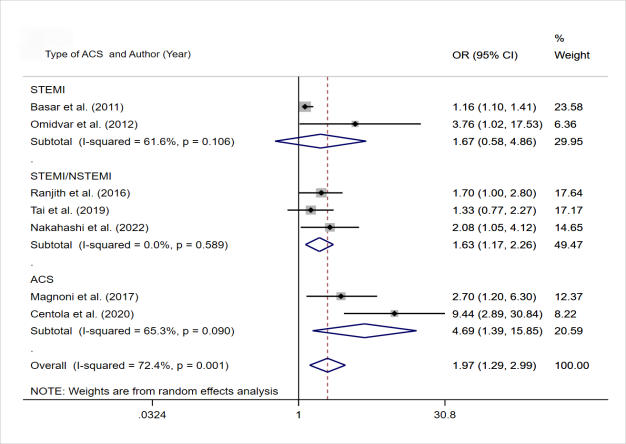** |
| Subgroup analysis of MACE(HR) | | |
| **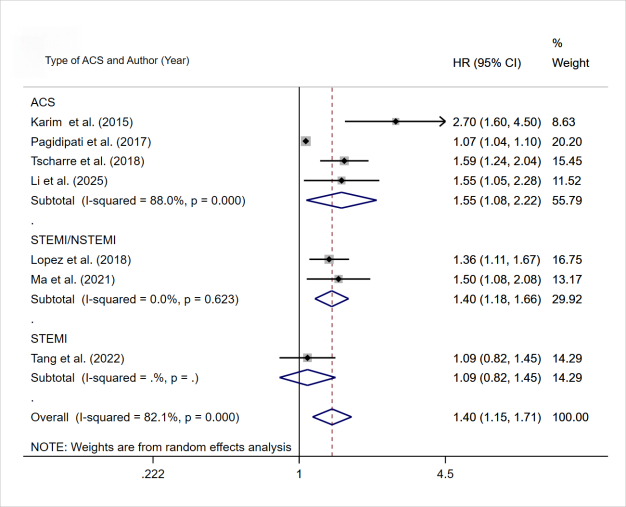** | | **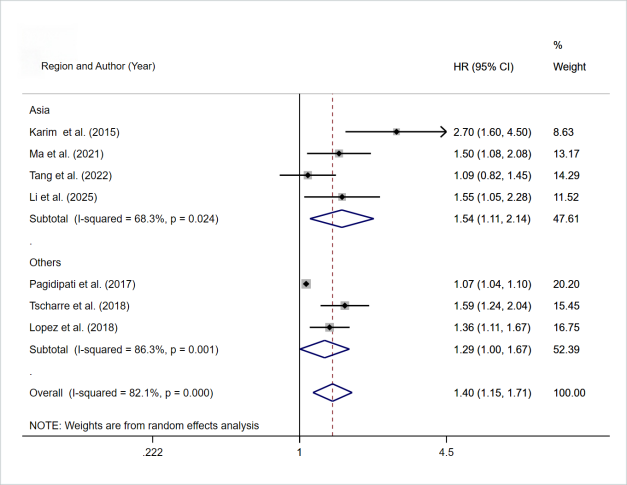** |
| Subgroup analysis of MACE(OR) | | |
| **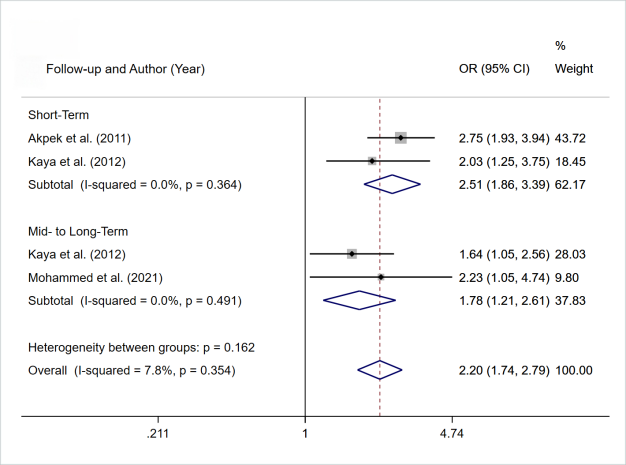** | | |
| Subgroup analysis of Cardiovascular Mortality | | |
| **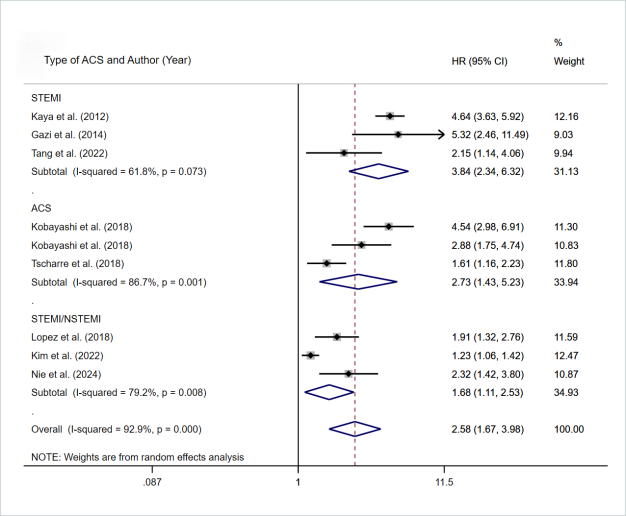** | | **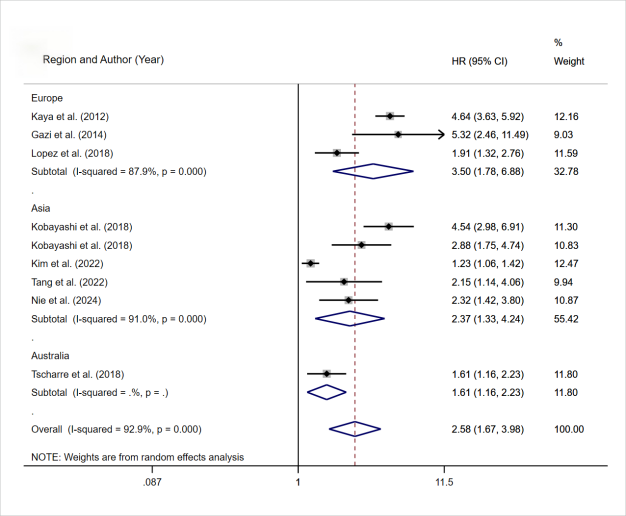** |
| **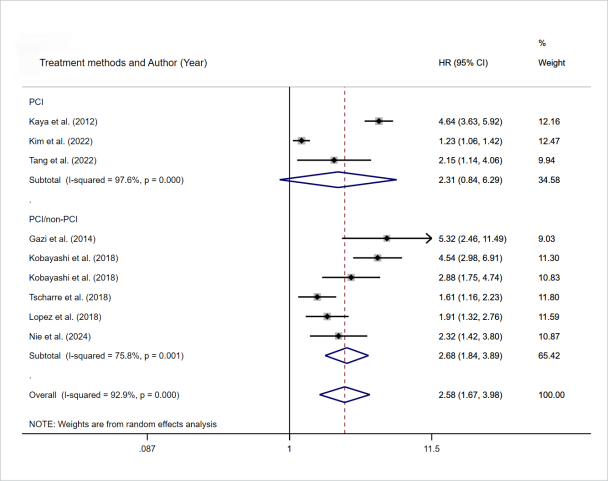** | | |
| Subgroup analysis of Revascularization | | |
| **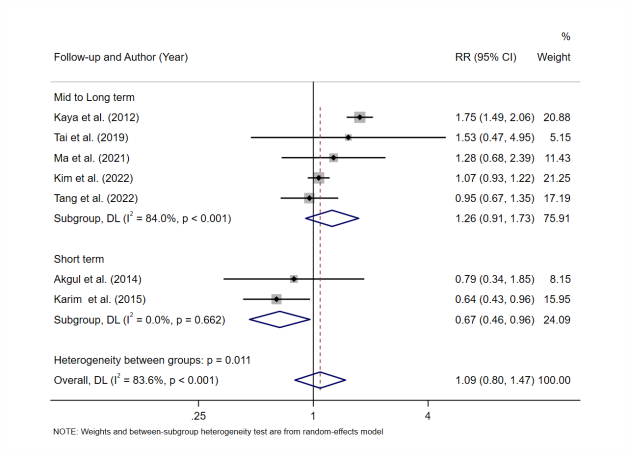** | | **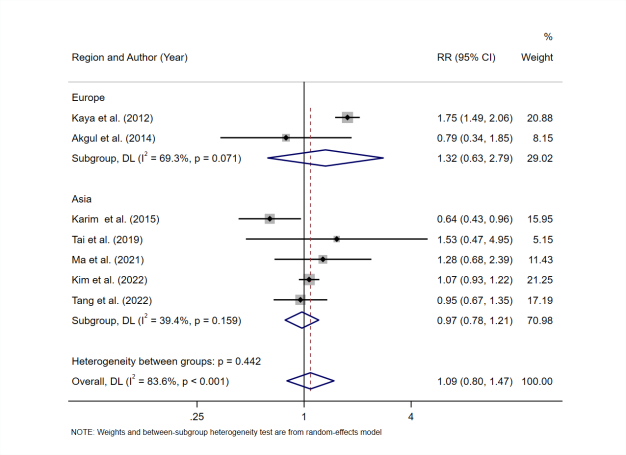** |
| **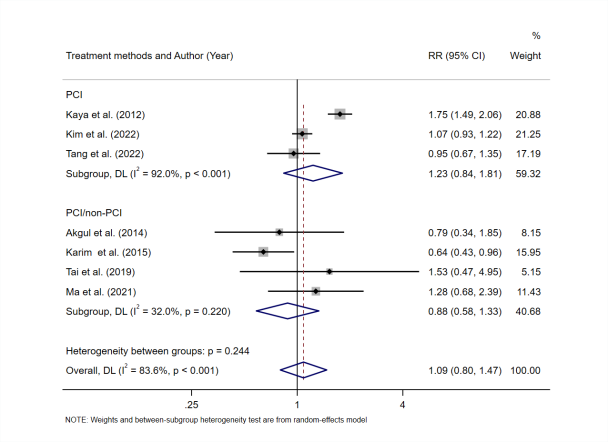** | | **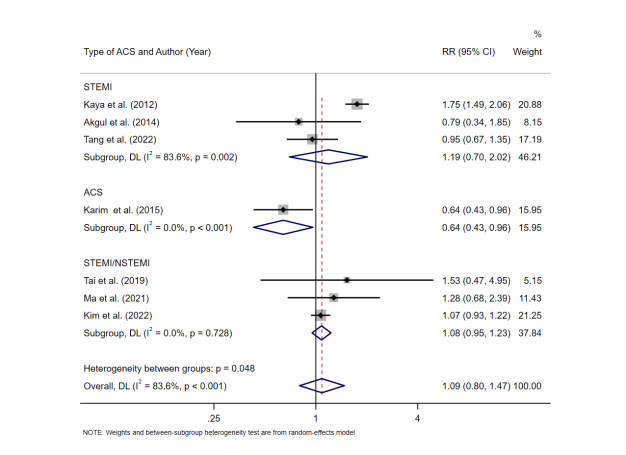** |
| Subgroup analysis of stroke | | |
| **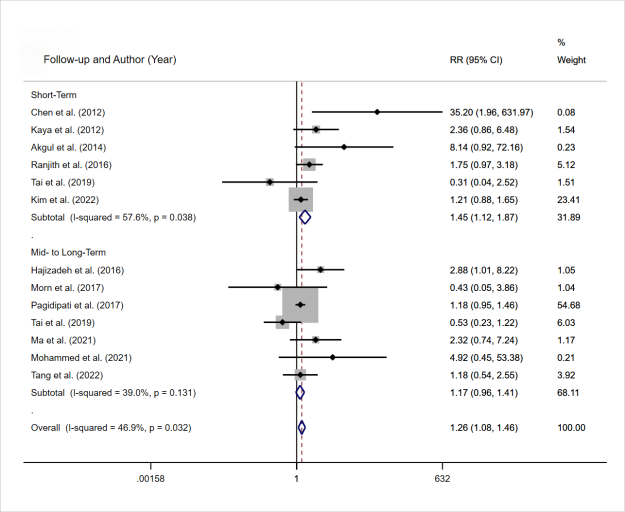** | **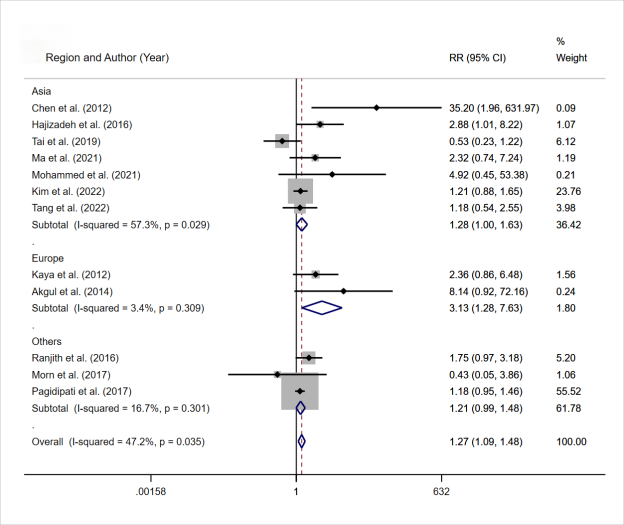** | |
| **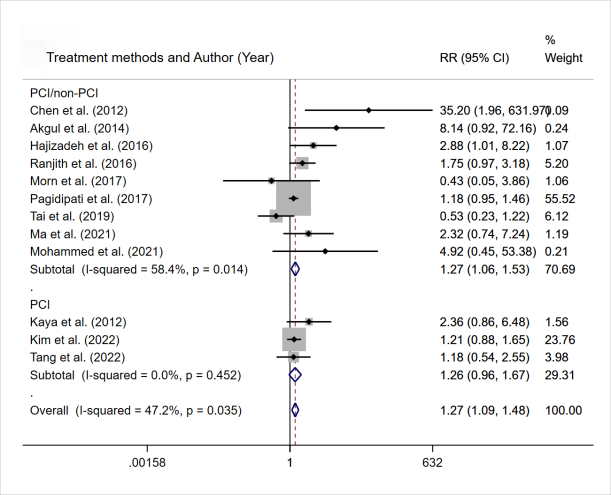** | **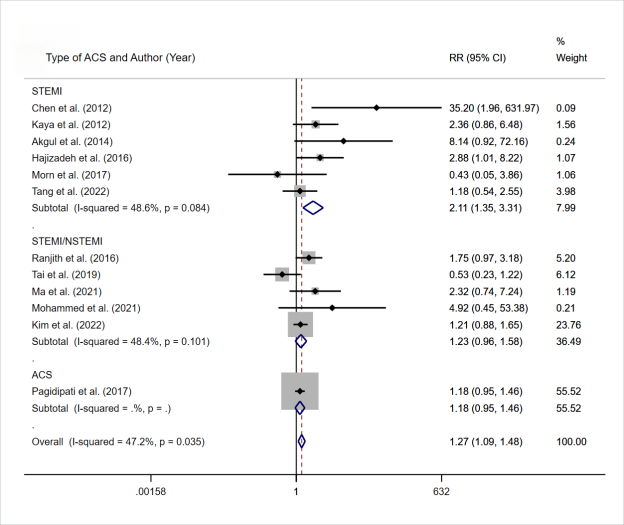** | |
| Subgroup analysis of heart failure | | |
| **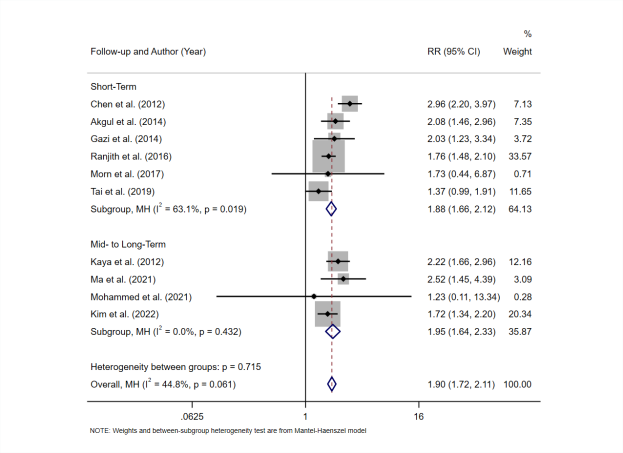** | **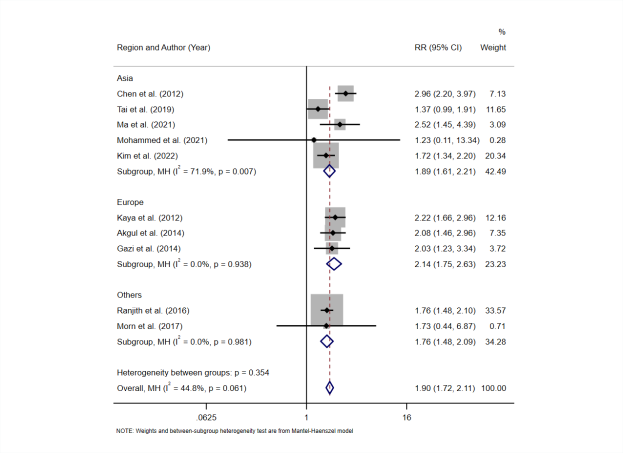** | |
| **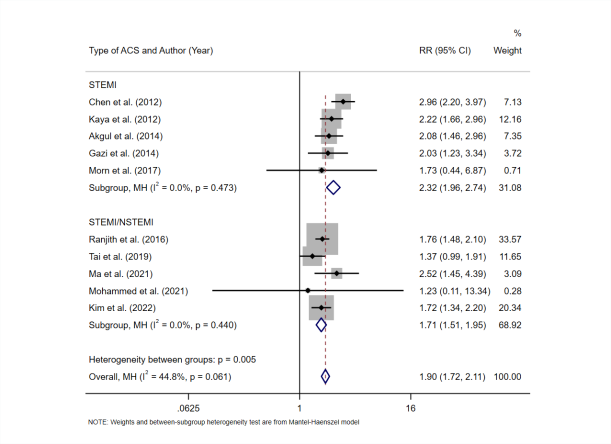** | **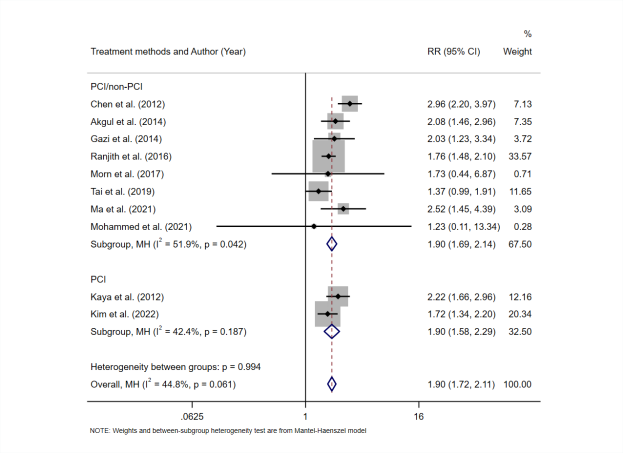** | |

**Supplementary Figure S2. Sensitivity analysis and publication bias analysis**

| All-Cause Mortality(HR) Egger: P＜0.001;Trim-and-fill method:P＜0.05 | | | |
| --- | --- | --- | --- |
|  | | | **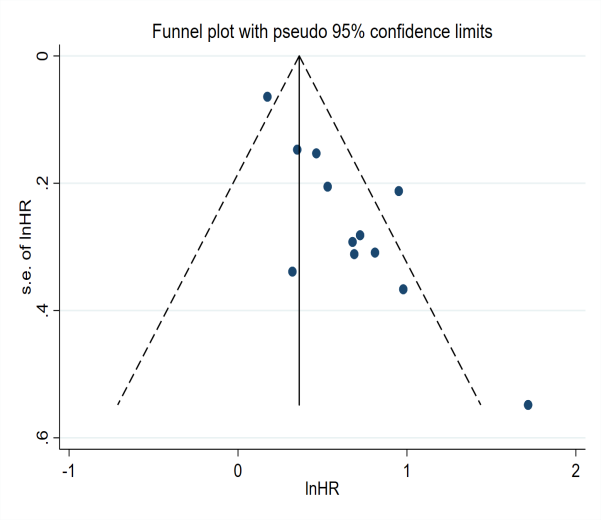** |
|  | | | |
| All-Cause Mortality(OR) | | | |
|  | | | |
| Mace(HR) | | | |
|  | | | |
| Mace(OR) | | | |
|  | | | |
| Cardiovascular Mortality | | | |
|  | |  | |
| Revascularization | | | |
|  | | | |
| **Stroke** Egger: P = 0.063 | | | |
|  | **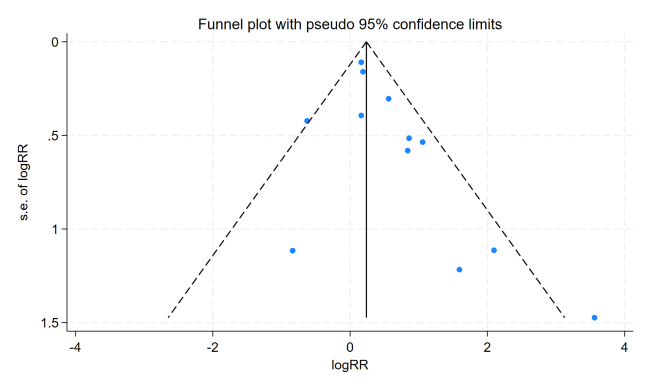** | | |
|  |  | | |
| **Heart failure** Egger: P = 0.712 | | | |
|  | | **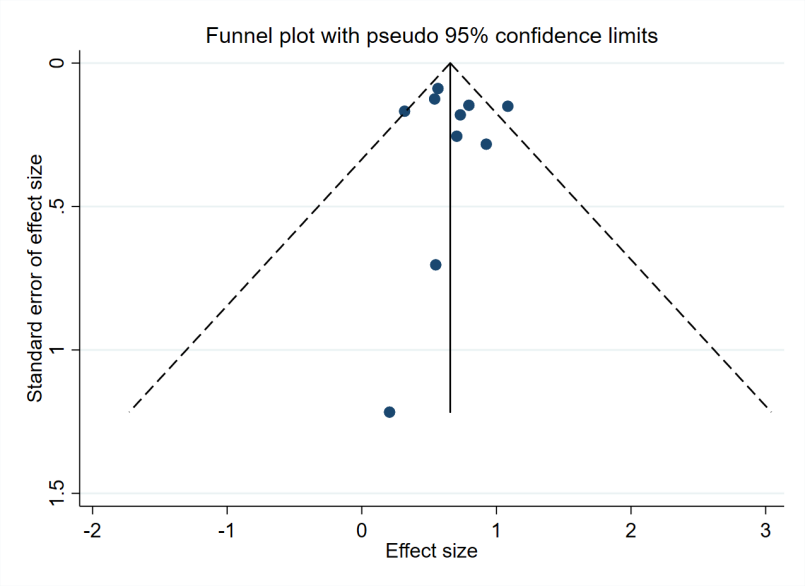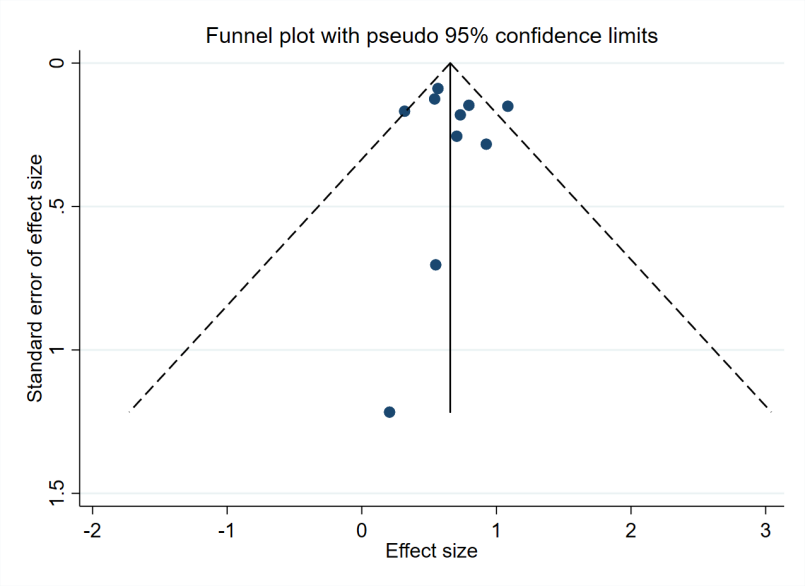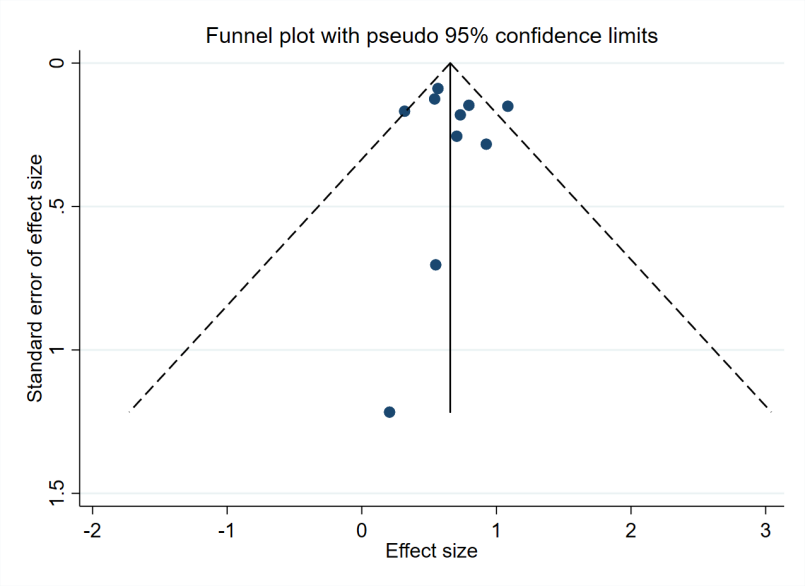** | |
